# Bringing seed barriers into focus: Optimized clearing and histological staining

**DOI:** 10.1111/tpj.71209

**Published:** 2026-11-03

**Authors:** Fabien Nirina Ramamonjy, Luca Piccinini, Vanessa Reyes Loaiza, Hongjun Meng, Robertas Ursache

**Affiliations:** ^1^ Centre for Research in Agricultural Genomics (CRAG), CSIC‐IRTA‐UAB‐UB Bellaterra Barcelona Spain

**Keywords:** ClearSeeα, developing seeds, seed coat cuticle, aliphatic barriers, histology, seed coat suberin, embryo cuticle, technical advance

## Abstract

Plant reproduction relies on tightly coordinated developmental programs that integrate environmental and endogenous signals. Within seeds, lipid‐based extracellular polymers such as cutin and suberin are deposited at defined apoplastic interfaces, where they function as selectively permeable barriers. These structures are actively remodeled by hormonal and metabolic cues to regulate hydration, nutrient exchange, and stress responses. Although the genetic and biochemical pathways underlying these barriers are increasingly well understood, their spatial organization and developmental dynamics remain difficult to investigate because they are embedded within optically dense and multilayered tissues. Existing clearing approaches have substantially improved visualization of internal seed structures, yet systematic whole‐mount imaging of multiple lipid‐based barriers across development, particularly in combination with fluorescent reporters, remains challenging. To address this, we developed an optimized pipeline that enables rapid and efficient seed tissue clearing and selective visualization of polyaliphatic barriers across four key seed interfaces: the seed surface, seed coat suberin, cuticle at the endosperm/seed coat boundary, and embryo surface cuticle. The method preserves the spatial context of these barriers within intact seeds and enables their co‐visualization with fluorescence‐based marker lines. Using a common whole‐mount imaging framework across successive developmental stages, we follow changes in the spatial organization of distinct barrier domains. The approach is also applicable to more complex seeds, such as those of *Camelina sativa,* and can be extended to other anatomically complex plant organs by combining the clearing and staining workflow with vibratome sectioning.

## Significance Statement

We present an optimized clearing and staining pipeline that enables high‐resolution imaging of cuticle and suberin barriers while preserving their spatial organization within intact developing seeds and allowing multiple barriers to be visualized across successive developmental stages using a common imaging framework. This approach reveals distinct developmental patterns of seed surface cuticle remodeling and seed coat suberin deposition and provides a scalable platform for three‐dimensional, functional, and comparative analyses of seed barriers.

## INTRODUCTION

Successful seed development depends on the establishment of several extracellular barriers that regulate permeability, protect against environmental stresses, and contribute to seed dormancy, longevity, and germination (De Giorgi et al., 2015; Debeaujon et al., 2000; Fedi et al., 2017; Hyvärinen et al., 2025; Renard et al., 2021). These structures include cuticles and suberin‐rich cell walls deposited at distinct interfaces between maternal and zygotic tissues, as well as specialized extracellular matrices that contribute to processes such as embryo development and seed germination (Doll et al., 2020; Gou et al., 2017; Hyvärinen et al., 2025; Ingram & Nawrath, 2017). Although their molecular composition and genetic regulation have been extensively studied, individual seed barriers have generally been characterized separately and using different experimental approaches. Consequently, integrating their spatial organization and relative developmental progression within a common experimental framework remains challenging.

Within *Arabidopsis thaliana* seeds, lipid‐based extracellular barriers are deposited at several distinct apoplastic interfaces (Figures 1a, 2a). The outermost seed surface is covered by a cuticle associated with the epidermal cells of the outer integument (Panikashvili et al., 2009; Watanabe et al., 2004). Beneath this layer, the oi1 layer of the seed coat undergoes extensive secondary cell wall modification and accumulates suberin and lignin. The importance of suberin accumulation in this layer to cope with osmotic, salt, and oxidative stress has been well documented (Beisson et al., 2007; Gou et al., 2017; Lashbrooke et al., 2016; Molina et al., 2008; Renard et al., 2021; Zhuang et al., 2025). At the boundary between maternal and zygotic tissues, the innermost integument layer (ii1) produces a cuticle that remains associated with the endosperm surface and contributes to the regulation of permeability and seed viability (Beeckman et al., 2000; Coen et al., 2019; De Giorgi et al., 2015; De Giorgi et al., 2021; Doll et al., 2020; Loubéry et al., 2018; Nawrath et al., 2013). In parallel, the developing embryo becomes enclosed by its own epidermal cuticle, which is essential for embryonic surface differentiation and integrity (González‐Valenzuela et al., 2023; Ingram & Nawrath, 2017; Moussu et al., 2013; Tanaka et al., 2001; Yang et al., 2008). Together, these structures establish a multilayered barrier network composed of cuticle‐ and suberin‐rich domains within the seed.

**Figure 1:**
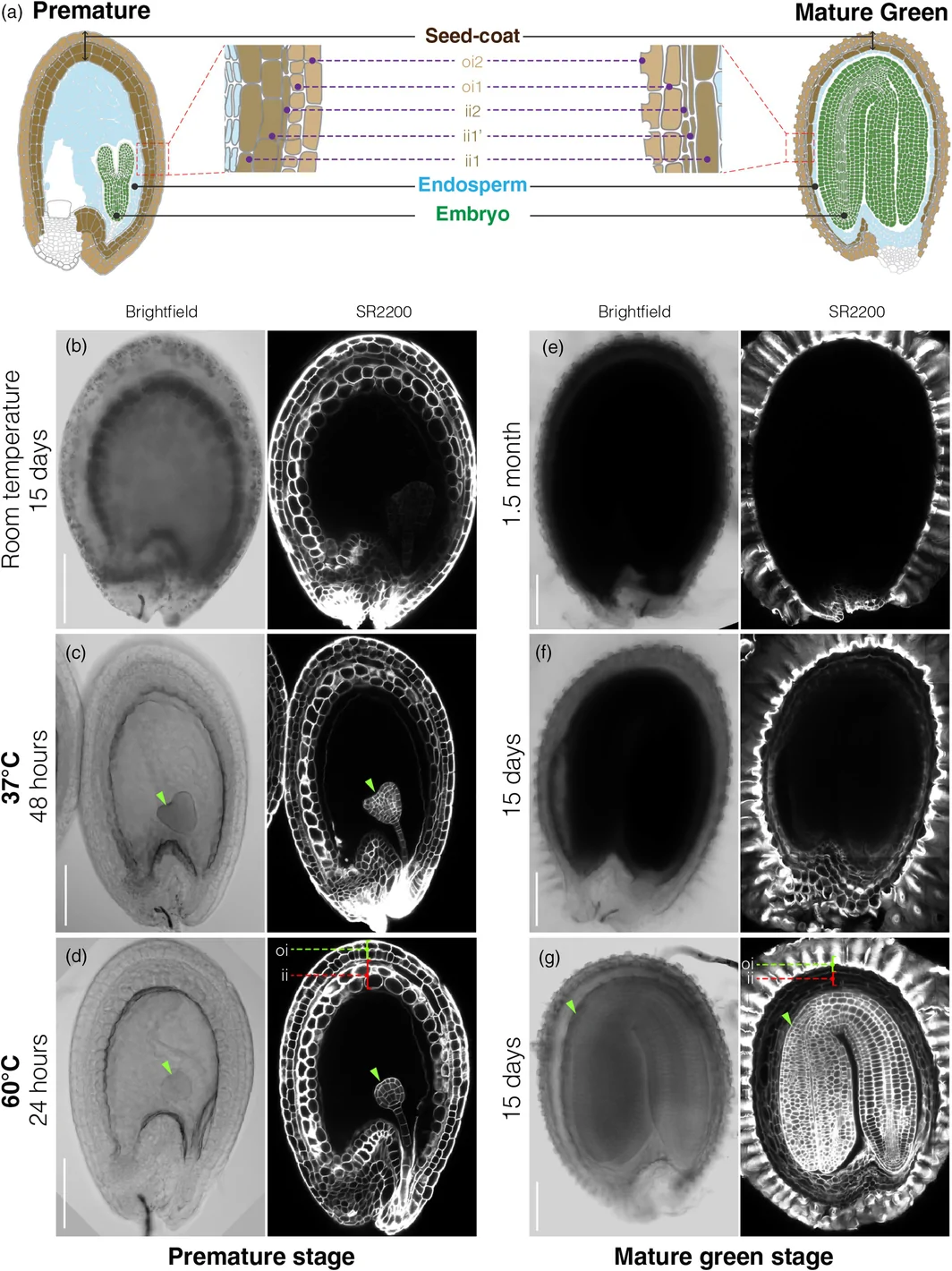
Moderate (37°C) or high (60°C) temperatures during Clearseeα incubation enhance whole‐seed clearing efficiency. (a) Anatomical representation of *Arabidopsis thaliana* seed tissues during development. The premature stage, represented here by the linear cotyledon stage, encompasses developmental stages from the preglobular to the bending cotyledon stage. Comparison of Clearseeα clearing supplemented with SR2200 in premature (b–d) and mature green (e–g) developing seeds at room temperature (b, e), 37°C (c, f), and 60°C (d, g). Panels b and c correspond to 3‐day post‐anthesis (DPA), whereas panel d corresponds to stages before 3DPA. Complete clearing of premature seeds was achieved after 48 h at 37°C or 24 h at 60°C (c, d), whereas mature green seeds (10 DPA) required 2 weeks at 60°C for complete clearing (g). oi: outer integument; ii: inner integument. Green arrows indicate the visible embryo. Scale bar: 100 μm.

**Figure 2:**
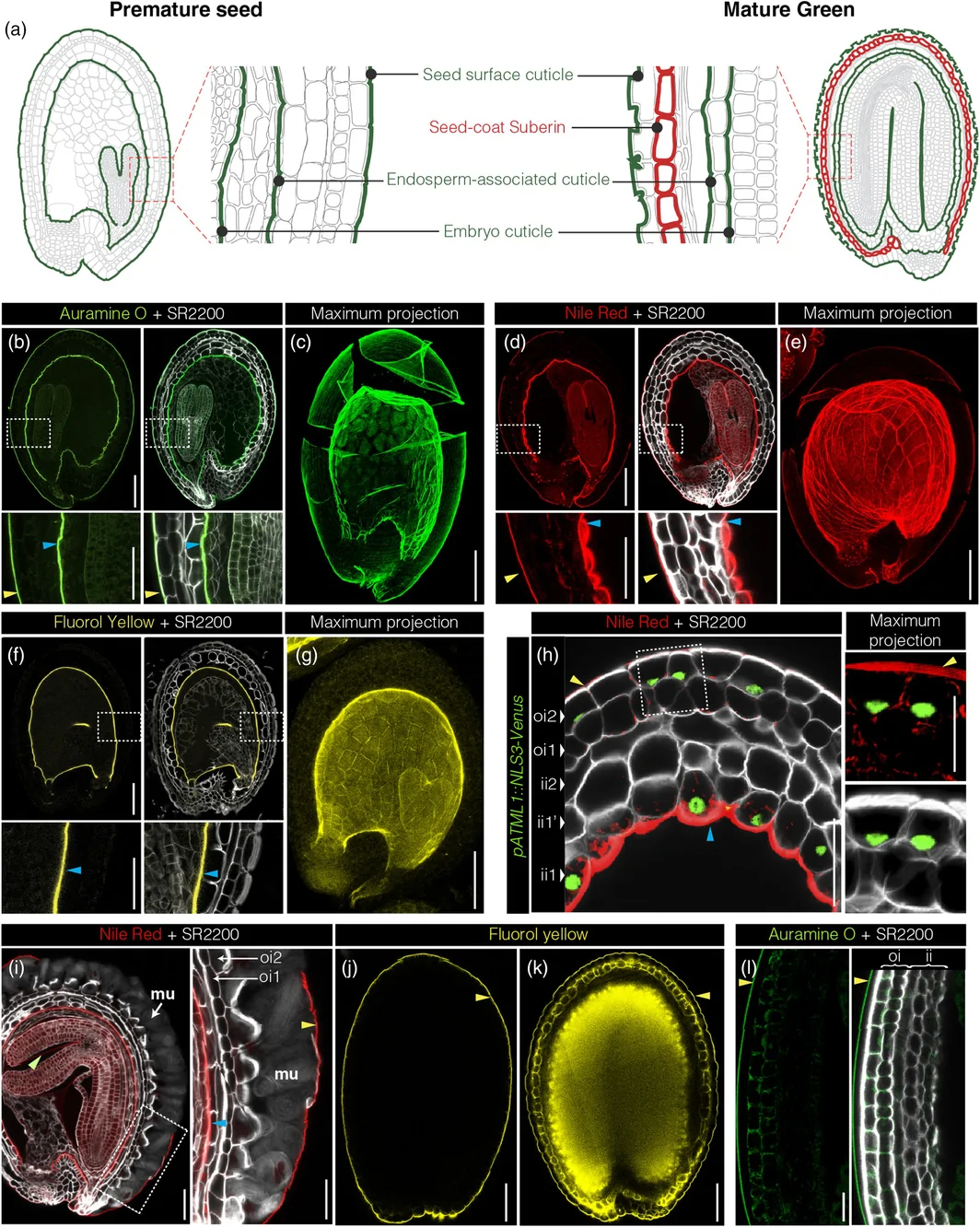
High‐temperature clearing enables high‐throughput, high‐resolution co‐visualization of cuticle‐abundant stains fluorescent reporters in premature Arabidopsis seeds. (a) Schematic representation of the localization of seed cuticular structures (green) and suberin (red) barrier in premature (left side) and mature green (right side) developing seeds. (b–i) Premature seeds are represented here by the heart, linear cotyledon and bending cotyledon stages, corresponding to 3, 5 and 7 DPA, respectively. (b) Linear cotyledon stage seed (5DPA) cleared at 60°C and co‐visualized with Auramine O and SR2200. (c) Maximum‐intensity projection of a linear cotyledon stage seed (5DPA) stained with Auramine O. (d) Linear cotyledon stage seed (5DPA) cleared at 60°C and co‐visualized with Nile Red and SR2200. (e) Maximum‐intensity projection of a linear cotyledon stage seed (5DPA) stained with Nile Red. (f) Heart stage seed (3DPA) cleared at 60°C and co‐visualized with Fluorol Yellow and SR2200. (g) Maximum‐intensity projection of a heart stage seed (3DPA) stained with Fluorol Yellow. (h) Co‐visualization of a premature seed (3DPA) with Nile Red and SR2200 together with the epidermal identity marker *pATML1::NLS‐3xVenus*. (i) Nile red‐stained late premature seed (bending cotyledon stage; 7 DPA) showing rupture of oi2 accompanied by mucilage extrusion and displacement of the seed surface cuticle. (j–k) Premature (3DPA; j) and mature green stage (10DPA; k) seeds cleared and stained using the methanol‐based Fluorol Yellow staining protocol, demonstrating preservation of seed surface cuticle while preventing mucilage extrusion. (l) Auramine O‐stained seed surface cuticle of a premature *Camelina sativa* seed cleared at 60°C for more than 15 days in Clearseeα supplemented with SR2200. Yellow and blue arrows indicate the seed surface and endosperm‐associated cuticle, respectively. oi: outer integument; ii: inner integument; mu: mucilage. Scale bar: 100 μm; close‐ups: 50 μm.

Previous studies have provided detailed insights into the composition, regulation, developmental appearance, and physiological functions of individual seed barriers, collectively establishing substantial knowledge of their spatial and developmental relationships. However, these barriers have generally been investigated separately and using different experimental approaches, making direct comparison of their organization and developmental dynamics challenging. A common imaging framework for systematic whole‐mount visualization of the major lipid‐based barrier domains across seed development is currently lacking. Such an approach would complement existing histological and ultrastructural studies by enabling their spatial organization and relative developmental progression to be examined under comparable experimental conditions while preserving their three‐dimensional context within intact seeds.

Several structures, including the embryo surface cuticle and the seed surface cuticle, have been characterized primarily using transmission electron microscopy, which provides exceptional ultrastructural resolution but requires extensive sample processing and provides information from selected two‐dimensional sections (González‐Valenzuela et al., 2023; Ingram & Nawrath, 2017; Panikashvili et al., 2009; Tanaka et al., 2001; Yang et al., 2008), making systematic comparison across multiple developmental stages and barrier domains laborious. Imaging of seed coat suberin presents additional challenges because the suberized oi1 layer is deeply embedded within the seed coat and becomes progressively compressed during maturation, limiting the accessibility of fixation, clearing, and staining reagents. Consequently, visualization of seed coat suberin has largely relied on electron microscopy or labor‐intensive histological approaches involving sectioning and microtome‐based processing prior to staining with suberin or cuticle stains, such as fluorescent Fluorol Yellow (FY), or non‐fluorescent Sudan red 7B stain (Beisson et al., 2007; Hyvärinen et al., 2025). Recently optimized optical clearing methods, including ClearSee, ClearSeeα, TDE, and FAST9, have substantially improved visualization of internal seed tissues, particularly during early developmental stages (Attuluri et al., 2022; Musielak et al., 2016; Kurihara et al., 2015, 2021). However, seeds at mature green stage remain difficult to image because of increasing tissue density, accumulation of pigments and storage compounds, strong autofluorescence, and limited stain penetration. As a result, comprehensive visualization of multiple lipid‐based barriers across seed development remains challenging.

Collectively, these barriers form a multilayered architecture extending from the seed surface to the embryo, providing a spatial framework for investigating barrier formation across successive tissue layers. Here, we establish an optimized ClearSeeα‐based clearing and imaging workflow that enables robust visualization of the major lipid‐based barrier systems within intact seeds. Using complementary fluorescent stains and reporter lines, we systematically resolve barrier domains across successive seed layers, progressing from the outer seed surface cuticle through seed coat suberin and the endosperm‐associated cuticle to the embryo cuticle. By applying a common imaging framework across developmental stages, the approach enables their spatial organization, remodeling, and relative developmental progression to be examined and compared within intact seeds. Furthermore, the workflow can be extended to structurally complex tissues through vibratome‐assisted sectioning.

## RESULTS

### Optimization of ClearSeeα clearing for efficient deep imaging of Arabidopsis seeds

Whole‐seed imaging remains challenging because the compact architecture of developing seeds restricts the penetration of fixation, clearing and staining solutions, and increases light scattering during image acquisition. To establish a workflow suitable for systematic whole‐mount imaging of seed barriers across developmental stages, we optimized ClearSeeα clearing conditions for Arabidopsis seeds and evaluated their ability to improve optical access to internal tissues (Figure 1b–g; Methods S1). Among the tested parameters, pre‐bleaching and alkaline washes did not improve overall clearing while maintaining compatibility with fluorescent imaging, whereas enzymatic pre‐treatment compromised tissue integrity. In contrast, temperature and vacuum‐assisted infiltration had the strongest positive impact on clearing efficiency. The combination of brief vacuum treatments during fixation and clearing with incubation at elevated temperatures substantially accelerated pigment removal and improved visualization of internal seed structures. In addition, we optimized sample mounting using a chambered cover glass and a thin layer of soft agarose gel, which improved seed shape preservation and its stability during imaging, resulting in more consistent acquisition of high‐resolution optical sections and three‐dimensional reconstructions (Methods S1; Figure S1). Using SCRI Renaissance 2200 (SR2200)‐labeled cell walls as a readout for stain penetration (Musielak et al., 2015), we observed a strong temperature dependence of clearing performance (Figure 1b–g). At room temperature, young seeds required up to 20 days of clearing and still retained dark deposits within the seed coat (Figure 1b). In contrast, incubation at 37°C reduced the clearing time to approximately 48 h (Figure 1c), while incubation at 60°C enabled visualization of internal tissues, including the embryo, within only 24 h, accompanied by substantial pigment reduction (Figure 1d). Seed developmental stage strongly influenced clearing efficiency. Whereas seeds from preglobular to bending cotyledon stages became readily transparent within 24–48 h under accelerated clearing conditions, mature green seeds remained substantially more recalcitrant (Figure 1e–g). Consistent with the accumulation of pigments, embryo greening, and seed coat development, seeds at the mature green stage maintained at room temperature remained opaque even after 6 weeks of clearing, limiting imaging to the outer integument layers (Figure 1e). Increasing the clearing temperature progressively improved optical accessibility, with partial visualization of internal tissues achieved after 2 weeks at 37°C (Figure 1f). Incubation at 60°C resulted in near‐complete clearing of seeds at the mature green stage within 2 weeks, allowing visualization of all major tissue layers, including the mature embryo, in both brightfield and fluorescence channels (Figure 1g). Together, these results establish a rapid clearing framework that reduces processing times from weeks to days and provides optical access to internal seed tissues across successive developmental stages, forming the basis for systematic whole‐mount visualization and comparison of lipid‐based barrier systems during seed development.

### Sequential visualization of lipid‐based barriers from the seed surface to the embryo

The Arabidopsis seed contains multiple lipid‐based barriers positioned at distinct apoplastic interfaces, extending from the seed surface to the embryo (Figure 2a). To resolve their spatial organization and developmental dynamics, we combined the optimized clearing conditions described above with complementary fluorescent stains and systematically examined barrier domains according to their natural arrangement within the seed. Using a common fixation, clearing, and imaging framework, we sequentially visualized the seed surface cuticle, seed coat suberin, the endosperm‐associated cuticle, and the embryo cuticle across successive developmental stages (Figures 2, 3, 4, 5; Methods S1). This outside‐to‐inside approach enables direct comparison of the spatial organization and developmental progression of barriers across successive tissue layers and provides, to our knowledge, the first systematic whole‐mount framework for layer‐by‐layer visualization of multiple lipid‐based apoplastic barriers across Arabidopsis seed development (Table 1). Across the developmental stages and staining conditions examined, staining was highly consistent, with all 113 seeds displaying the expected cuticle and/or suberin staining patterns (Figure S2). The main source of technical variability was associated with preservation of the seed surface cuticle during sample mounting rather than staining efficiency (Figure S1).

**Table 1:** Seed barrier and stain dependant optimal workflow guide detailing solutions used during procedure and fluorescent protein compatibility

Seed apoplastic barrier	Developmental stage	1‐Fixation/Washing	2‐Clearing method	Optimal Stains	3‐Staining medium	4‐Stain washing medium	Supplementary protocol	Fluorescent protein compatibility
Seed surface cuticle	Globular to linear cotyledon	4% PFA/PBS	37°C/60°C ClearSeeα	AO	ClearSeeα	ClearSeeα	A‐1	Yes
				NR			A‐2	
	Bending cotyledon to Mature green	Methanol		FY	Methanol	Methanol	B‐1	No
Endosperm‐associated seed coat cuticle	Globular to Mature green	4% PFA/PBS	37°C/60°C ClearSeeα	AO	ClearSeeα	ClearSeeα	A‐1	Yes
				NR			A‐2	
				FY	Ethanol	Ethanol:Water gradient	A‐3	No
Embryo cuticle	Globular to Bending cotyledon	4% PFA/PBS	37°C/60°C ClearSeeα	AO	ClearSeeα	ClearSeeα	A‐1	Yes
				NR			A‐2	
				FY	Ethanol	Ethanol:Water gradient	A‐3	No
	Mature green			FY				
Seed coat suberin	Mature green	4% PFA/PBS	37°C/60°C ClearSeeα	AO	ClearSeeα	ClearSeeα	A‐1	Yes
				NR		Water	A‐2	
				FY	Ethanol	Ethanol:water gradient	A‐3	No
	Mature dry	Methanol		FY	Methanol	Methanol	B‐1	No
				NR			B‐2	

### Visualizing the seed surface cuticle

The seed surface cuticle associated with the epidermal cells of the outer integument has been previously described and implicated in maintaining seed surface integrity (Panikashvili et al., 2009; Watanabe et al., 2004). However, its organization, continuity, and remodeling across seed development remain poorly understood. Here, our optimized pipeline enables visualization of this barrier in intact seeds across developmental stages, revealing previously inaccessible aspects of its dynamics. During differentiation into mucilage secretory cells, epidermal cells undergo extensive cell wall remodeling, likely leading to temporal redistribution or partial loss of the cuticle (Ingram & Nawrath, 2017; Western et al., 2000). To capture these dynamics, SR2200‐prestained seeds were incubated in ClearSeeα containing Nile Red (NR) or Auramine O (AO) at 37°C for 12 h (preglobular to bending cotyledon stages) or 24 h (mature green). NR has previously been used to visualize cutin‐ and suberin‐containing structures in different plant tissues and is compatible with ClearSee‐based imaging and fluorescent protein reporters (Piccinini et al., 2024; Ursache et al., 2018). Here, we applied NR within the optimized whole‐mount seed clearing workflow to examine lipid‐based barriers across seed development. The staining at 37°C allowed for a preservation of cuticle integrity while allowing sufficient and rapid stain penetration (Methods S1; Table 1). Both NR and AO strongly labeled the surface cuticle of young seeds (globular to linear cotyledon stages), outlining the outer integument 2 (oi2) cell wall (Figure 2b–e). This pattern is consistent with previous observations of the cuticular layer associated with the outer integument epidermis (Panikashvili et al., 2009; Watanabe et al., 2004), supporting the ability of the whole‐mount staining approach to resolve this barrier. During maturation, however, we observed progressive changes in organization and continuity of this layer. From the bending cotyledon stage onward, mucilage deposition coincided with accumulation of SR2200‐positive material, rupture of mucilage secretory cells, and extrusion of mucilage, accompanied by displacement or partial loss of the surface cuticle (Figure 2i; Figure S3). In later stages (mature green), this resulted in exposure of columellae and seed surface cuticle displacement (Figure 3a–e). This observation suggests dynamic remodeling of oi2 cell wall during seed maturation and the provided methodology might serve as a tool to describe this process (Ingram & Nawrath, 2017; Western et al., 2000). To assess the compatibility of NR for the surface cuticle, we combined it with a reporter for ARABIDOPSIS THALIANA MERISTEM LAYER1 (ATML1), an HD‐ZIP class IV transcription factor and a master regulator of epidermal cell identity (Iida et al., 2019; Takada et al., 2013; Takada & Jürgens, 2007). Using our protocol, we cleared the line expressing *pATML1::NLS‐3xVenus* and combined with NR and SR2200 stains. We observed NLS‐3xVenus signal abundance in the oi2, together with the surface cuticle and SR2200 outlined cell walls (Figure 2h). Basic Fuchsin staining, commonly used for visualization of lignified cell walls (Piccinini et al., 2024), was also compatible with the ClearSeeα‐based procedure and produced a distinct signal at the seed surface (Figure S4e,f). Whether the Basic Fuchsin‐derived signal at the seed surface reflects lignin or phenolic components associated with the cuticle remains to be determined.

**Figure 3:**
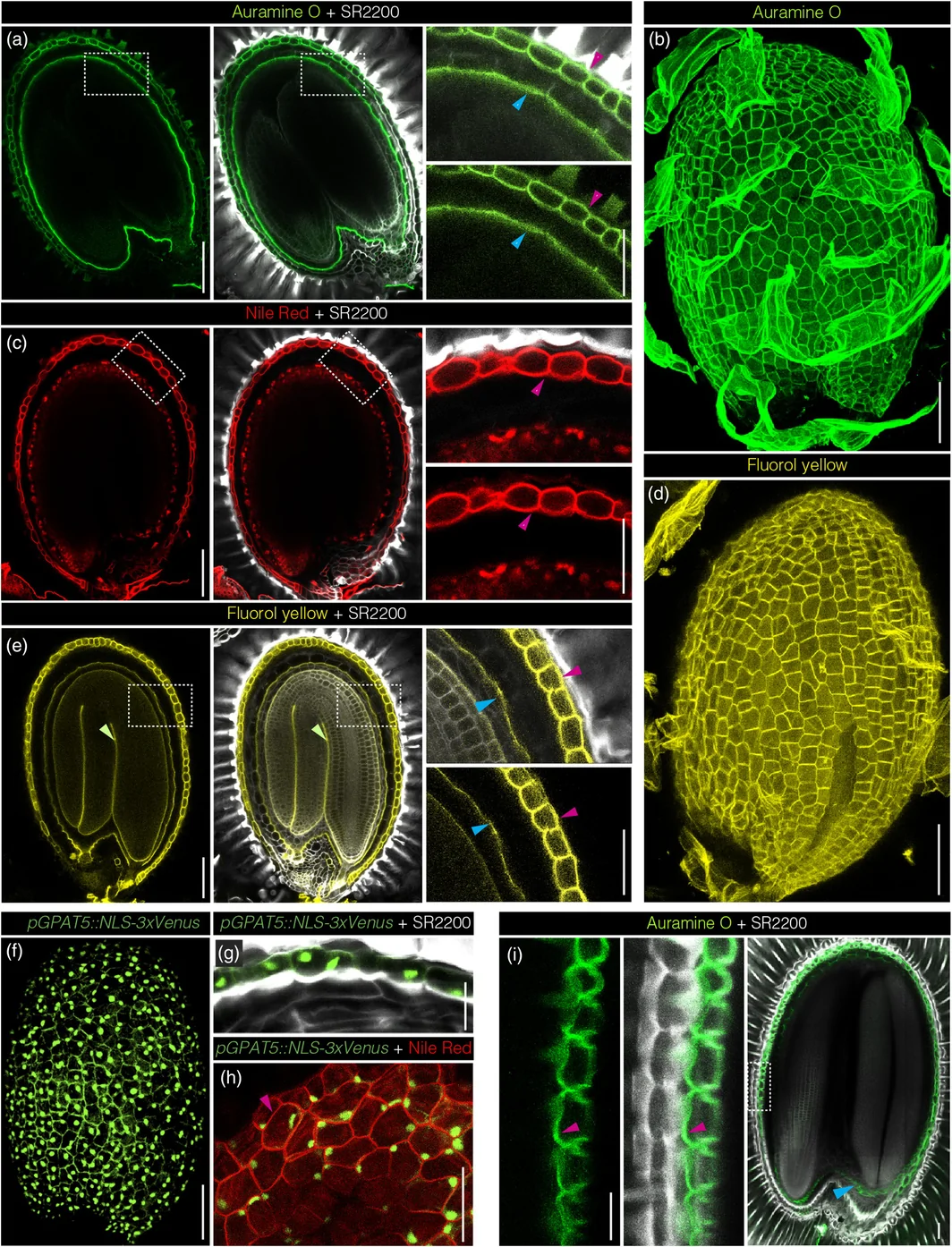
High‐temperature clearing enables high‐resolution co‐visualization of seed coat suberin with fluorescent stains and fluorescent markers in mature green seeds of *Arabidopsis thaliana* (a–h) and *Camelina sativa* (i). (a–e) Mature green Arabidopsis seeds (10 DPA) processed using the optimized clearing protocol and stained with Auramine O and SR2200 (a, b), Nile Red and SR2200 (c), or Fluorol Yellow and SR2200 (d, e), highlighting suberin deposition in the subepidermal layer of the seed coat. Panels a, c and e show single optical sections, whereas b and d show maximum‐intensity projections. (f–h) Expression of *pGPAT5::NLS‐3xVenus* coinciding with suberin deposition in the outer integument 2 (oi2), shown as a maximum‐intensity projection (f), and in combination with SR2200 (g) or Nile Red (h). (i) Auramine O‐stained seed coat suberin in a mature green *Camelina sativa* seed cleared at 60°C for more than 1.5 months in Clearseeα supplemented with SR2200. Pink, green and blue arrows indicate seed coat suberin, embryo and endosperm‐associated cuticles, respectively. Scale bar: 100 μm, close‐up = 50 μm.

Ethanol‐based FY staining, on the other hand, following ClearSeeα clearing failed to preserve the surface cuticle (Figure 2f,g), likely due to the exposed nature of the cuticle and sensitivity to transitions between solvents of different viscosity (Methods S1). To overcome this limitation, we implemented the previously published methanol‐based protocol for fixation, clearing, and FY staining (Ursache et al., 2021), with a modification that involved extending the FY staining step to overnight instead of 1 hour. This modification enabled consistent preservation and visualization of the seed surface cuticle across the developmental stages examined (Figure 2j,k). To assess the broader applicability of the method, we applied the protocol to *Camelina sativa* (Camelina) seeds, which are larger and structurally more complex than Arabidopsis. Similar to Arabidopsis, Camelina exhibited a prominent seed surface cuticle at early developmental stages (Figure 2l; Figure S5a).

### Imaging seed coat suberin in intact seeds

Seed coat suberin has previously been localized to the outer integument 1 (oi1) layer, with its accumulation reported during late seed development (Hyvärinen et al., 2025). Using our whole‐mount imaging pipeline, we followed the appearance and spatial organization of this barrier across a developmental series of intact seeds. Seed coat suberin was first detected during the transition from bent cotyledon to mature green stages and remained confined to the outer integument 1 (oi1) layer throughout development (Figure 3a–e). Imaging this barrier is particularly challenging because the suberized oi1 layer is embedded within the seed coat and becomes progressively compressed during maturation, restricting accessibility for clearing and staining reagents.

To visualize seed coat suberin, we combined the optimized clearing procedure with three fluorescent dyes previously used to label extracellular suberin barriers: FY, NR, and AO (Piccinini et al., 2024; Ursache et al., 2018). All three stains consistently labeled the oi1 layer of the seed coat (Figure 3a–e). This localization is consistent with previous histological, ultrastructural, and biochemical characterization of suberin deposition in the oi1 layer (Beisson et al., 2007; Gou et al., 2017; Hyvärinen et al., 2025; Molina et al., 2008; Renard et al., 2021), supporting the ability of the whole‐mount staining approach to resolve the previously characterized seed coat suberin barrier. Notably, suberin deposition was not detected during early embryogenesis but became clearly visible during the transition from bent cotyledon to mature green stages (Figure 3a–d; Figure S3a–h), consistent with the developmental timing reported by Hyvärinen et al. (2025). Expression of the suberin‐associated reporter *pGPAT5::NLS‐3xVenus*, marking the activity of GLYCEROL‐3‐PHOSPHATE ACYLTRANSFERASE 5 (GPAT5), a key enzyme involved in suberin monomer biosynthesis (Beisson et al., 2007), coincided with the stained oi1 layer, further supporting the developmental specificity of suberin accumulation and compatibility of the technique with fluorescent proteins (Figure 3f–h). Importantly, seed coat suberin remained readily detectable by Nile Red staining in mature dry seeds (Figure 4), demonstrating that the method can visualize the suberin barrier both during its establishment in late seed maturation and after completion of seed development. Interestingly, Basic Fuchsin staining, commonly used for lignin visualization (Piccinini et al., 2024), also produced a strong signal in the same outer integument 1 (oi1) layer in which suberin accumulates in the seed of mature green stage (Figure S4e,f). This demonstrates the compatibility of our clearing procedure with Basic Fuchsin staining and highlights the co‐localization of aliphatic and phenolic barrier components within this seed coat layer. Similar to Arabidopsis, Camelina exhibited a prominent seed coat suberin layer that became detectable at late developmental stages (Figures 2l and 3i; Figure S5a,b). Together, these results establish a framework for high‐resolution visualization of seed coat suberin deposition and provide direct insight into its spatial organization and developmental progression within intact seeds.

**Figure 4:**
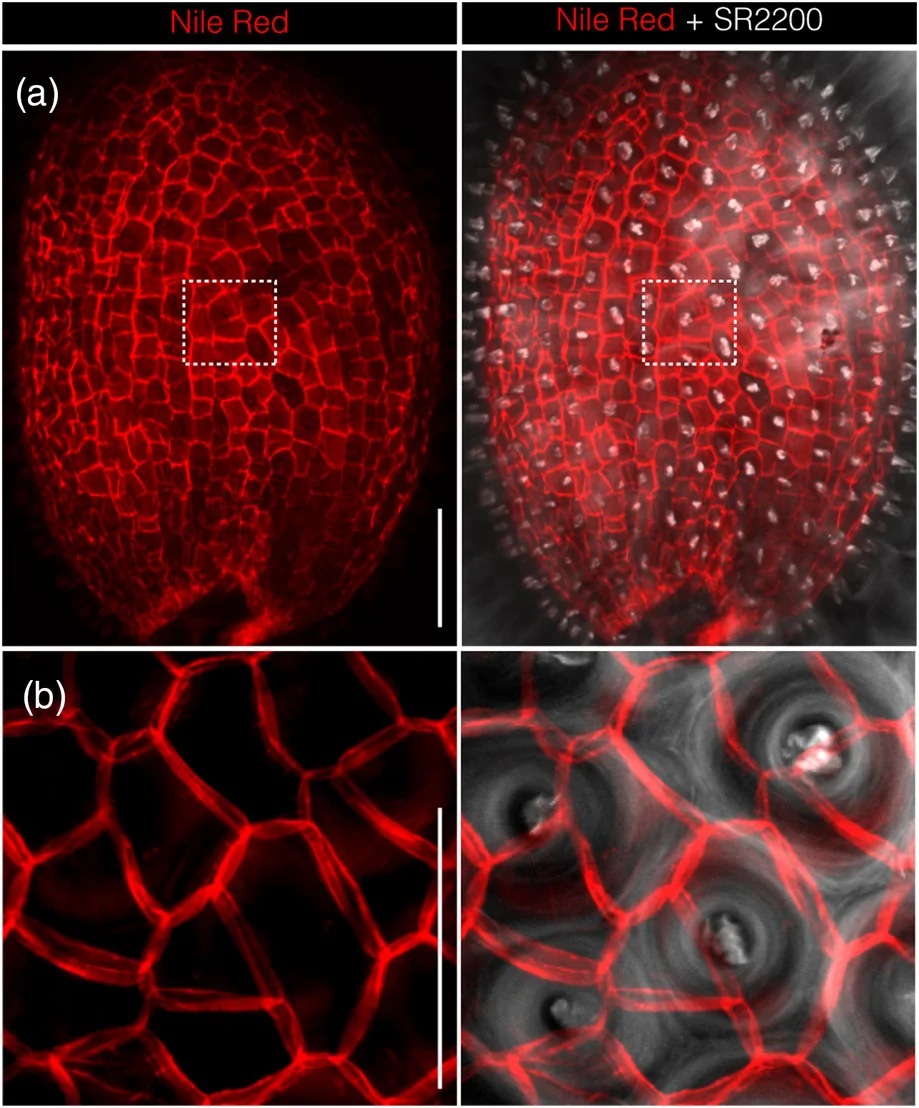
High‐resolution co‐visualization of *Arabidopsis thaliana* mature dry seed suberin with Nile Red and SR2200 in. (a, b) Maximum‐intensity projection representation of whole dry seed cleared and stained following the methanol‐based Nile Red staining protocol. Scale bar: 100 μm (a), 50 μm (b).

### Revealing the endosperm‐associated cuticle

The endosperm‐associated cuticle is embedded between tightly packed tissues, while seeds at the mature green stage are increasingly optically opaque and autofluorescence from proanthocyanidins in the seed coat interferes with signal detection. Previous studies have visualized this barrier using transmission electron microscopy, providing detailed information on its ultrastructure, as well as light microscopy approaches using histological dyes to examine its localization and organization (Coen et al., 2019; De Giorgi et al., 2015; Demonsais et al., 2020; Loubéry et al., 2018). These approaches have established important features of the endosperm‐associated cuticle but generally rely on sectioned material, making systematic analysis of its three‐dimensional organization within intact seeds challenging.

Applying our optimized ClearSeeα‐based pipeline, we visualized the endosperm‐associated cuticle in intact seeds using the same fixation, clearing, and staining framework described above (Figures 2b–h and 3a,e; Methods S1). In developing seeds up to the bending cotyledon stage, this layer was clearly labeled by AO, NR, and FY (Figure 2b–h). The position and overall organization of the stained layer are consistent with previous histological and ultrastructural descriptions of the endosperm‐associated cuticle (Coen et al., 2019; De Giorgi et al., 2015; Demonsais et al., 2020; Loubéry et al., 2018), supporting the ability of the whole‐mount approach to resolve this previously characterized barrier. The whole‐mount approach further enabled examination of the three‐dimensional organization of the endosperm‐associated cuticle at the chalazal region, where multiple maternal and zygotic tissues converge. Three‐dimensional reconstructions revealed the spatial arrangement of the cuticular interface around this structurally complex region, including the localized discontinuity at the chalazal end previously described by Loubéry et al. (2018), without the need for physical sectioning (Figure S6). This demonstrates an advantage of whole‐mount imaging for resolving barrier organization at seed interfaces that can be difficult to reconstruct from individual sections.

Basic Fuchsin staining was also compatible with the clearing procedure and produced a signal at the same endosperm‐associated interface (Figure S4e,f). In mature green stage seeds, however, clear and consistent labeling of the endosperm‐associated cuticle was observed primarily with AO and FY (Figure 3a,e), whereas NR revealed droplet‐like structures in the endosperm (Figure 3c), consistent with reported accumulation of intracellular lipids in developing endosperm (Miray et al., 2021).

We observed the presence of the epidermal cell identity marker, *pATML1::NLS‐3xVenus*, in ii1 layer, coinciding with the endosperm‐associated cuticle (Figure 2h). Together, these results demonstrate that the developed procedure enables rapid whole‐mount visualization of the endosperm‐associated cuticle while preserving its spatial context within intact seeds and compatibility with fluorescence‐based genetic markers.

### Visualizing embryo surface cuticle formation

The embryo surface cuticle undergoes dynamic formation and maturation during embryogenesis, but its spatial organization within intact developing seeds remains difficult to examine. Imaging this structure within intact seeds is particularly challenging because the embryo is deeply embedded within surrounding tissues, restricting accessibility for fixation, clearing, and staining. Consequently, most studies of embryo cuticle organization have relied on electron microscopy, which provides exceptional ultrastructural resolution but requires extensive sample processing and provides information from selected two‐dimensional sections (Stępiński et al., 2017; Stępiński et al., 2020; Tanaka et al., 2001; Yang et al., 2008). The embryo cuticle has also been visualized using Auramine O staining, but this approach required embryos to be dissected from living ovules prior to staining and imaging (Szczuka & Szczuka, 2003).

Using our optimized clearing pipeline, we visualized the embryo cuticle across several developmental stages within intact seeds using FY, AO, and NR (Figure 5; Table 1; Methods S1). To improve FY imaging of embryo cuticle, we applied an ethanol gradient‐based wash following staining, which enhanced preservation of embryo morphology during sample preparation (Methods S1). FY provided strong and continuous labeling of the embryo surface from globular to mature green stages (Figures 3e, 5c). AO and NR effectively labeled the young embryo cuticle while preserving embryo integrity (Figure 5a,b,d,e). The localization and continuity of the stained layer at the embryo surface are consistent with previous Auramine O‐based observations of dissected embryos (Szczuka & Szczuka, 2003) and with ultrastructural characterization of the embryonic cuticle (Stępiński et al., 2017; Tanaka et al., 2001; Yang et al., 2008). Importantly, the present approach enables this barrier to be visualized within the intact cleared seed without prior embryo dissection, preserving its spatial relationship with the surrounding seed tissues.

**Figure 5:**
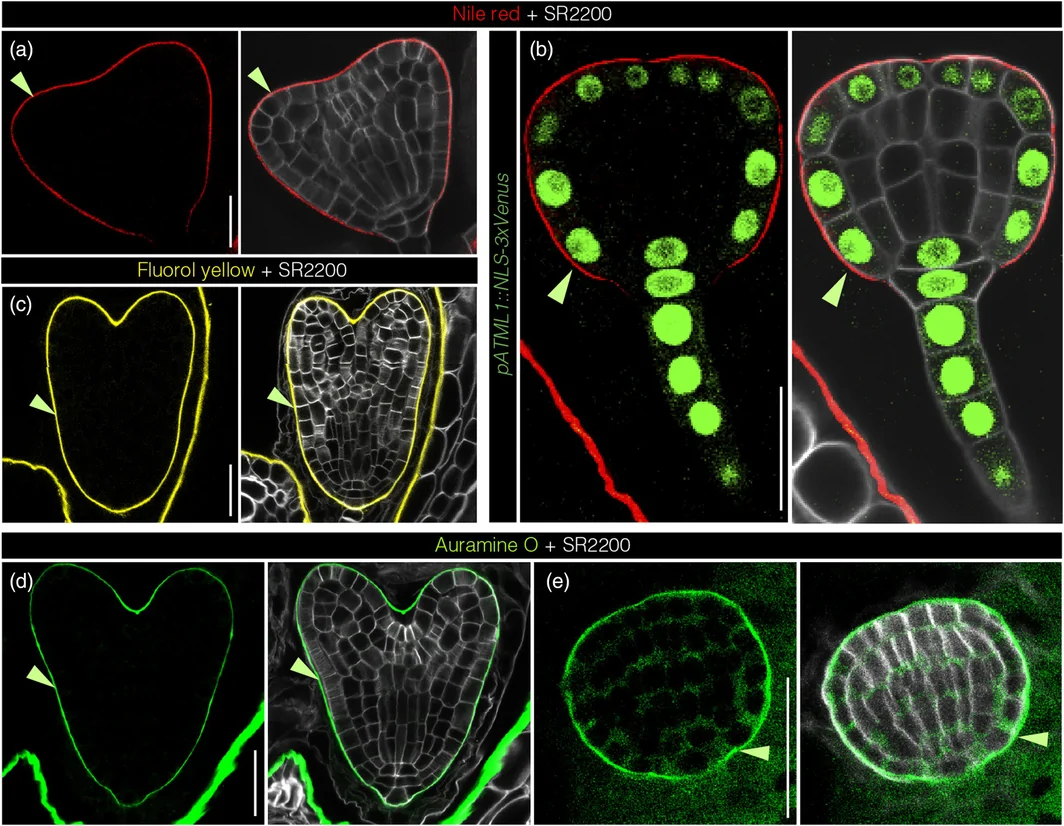
High‐temperature clearing enables rapid, high‐resolution visualization of early embryo cuticle development using fluorescent stains and fluorescent reporters. (a, b) Nile red staining of embryo cuticle in wild type (a) and *pATML1::NLS‐3xVenus* expressing (b) Arabidopsis seeds at the heart stage and late globular stages (3 DPA), respectively. (c) Fluorol yellow staining, using the ethanol‐based protocol, of the embryo cuticle in a heart‐stage Arabidopsis seed (3 DPA). (d) Auramine O staining of the embryo cuticle in a heart‐stage Arabidopsis seed (3 DPA). (e) Auramine O staining of the embryo cuticle in a globular‐stage *Camelina sativa* seed, co‐visualized with SR2200. Samples were stained following Clearseeα incubation at 60°C for 24 h for premature Arabidopsis seeds (a–d) and 5 days for the more structurally complex premature Camelina seeds (e). Green arrows indicate the embryo cuticle. Scale bar: 25 μm.

Consistent with previous reports, the cuticle was restricted to the embryo proper and was absent from the suspensor (Ingram & Nawrath, 2017; Szczuka & Szczuka, 2003) (Figure 5a,b).

The epidermal cell identity reporter *pATML1::NLS‐3xVenus* was also detected in the epidermal layer of the embryo proper, consistent with previous reports of ATML1 expression (Iida et al., 2019; Takada & Jürgens, 2007) (Figure 5b). Together, these results demonstrate that the developed procedure enables whole‐mount visualization of the embryo surface cuticle across successive developmental stages while preserving its spatial relationship with surrounding seed tissues and compatibility with fluorescence‐based genetic markers.

### Cell wall barrier architecture revealed by sectioning‐assisted optical clearing

Although optical clearing substantially improves visualization of internal tissues, whole‐organ imaging remains challenging in structurally complex samples such as developing siliques, mature leaves, and large seeds. In these tissues, limited reagent penetration, tissue autofluorescence, and optical scattering can restrict visualization of internal barrier structures. To overcome these limitations, we combined vibratome sectioning with the optimized ClearSeeα‐based clearing and staining workflow (Figure 6a; Figure S7). Sectioning substantially improved tissue accessibility and enabled efficient clearing and staining of thick, multilayered samples while preserving overall tissue architecture.

**Figure 6:**
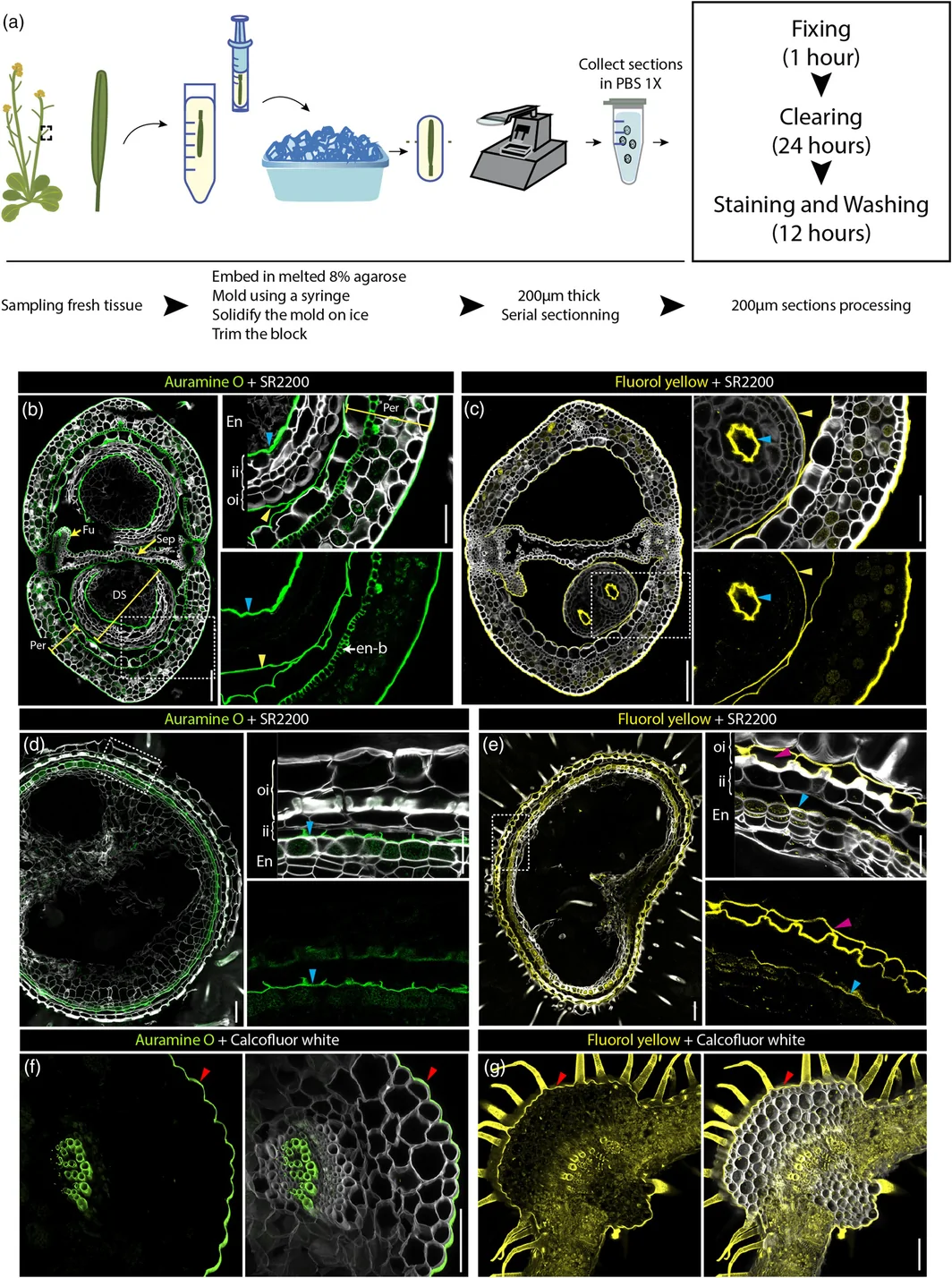
Combining the optimized clearing and staining procedure with vibratome sectioning enables high‐throughput, high‐resolution imaging of cuticle and suberin barrier across diverse tissues and species. (a) General workflow combining vibratome sectioning with rapid fixation, clearing and staining. (b, c) Cleared transverse sections of young Arabidopsis siliques stained with Auramine O (b) and Fluorol Yellow (c), revealing the seed surface and endosperm‐associated cuticles, inner and outer pericarp cuticle‐like structures, as well as lignified endocarp‐b cells (b). (d, e) Premature (d) and mature green (e) *Camelina sativa* seeds stained with Auramine O and Fluorol Yellow, respectively, showing developmental stage‐dependent deposition of seed coat suberin at the mature green stage. (f, g) Cleared and stained leaf sections of 3 weeks old *Arabidopsis thaliana* (f) and 9 days old *Solanum lycopersicum* (g) stained with Auramine O (f) or using the methanol‐based Fluorol Yellow staining protocol (g). Samples were co‐stained with SR2200 (b–d) or Calcofluor White (e, f) to outline cell walls. Yellow, blue, pink and red arrows indicate the seed surface, endosperm‐associated cuticle seed coat suberin and leaf cuticle, respectively. Fu: funiculus, Sep: septum, DS: developing seed, Per: pericarp, En: endosperm, ii: inner integument, oi: outer integument and en‐b: endocarp‐b cells. Scale bars: 100 μm (a–e), 50 μm (close‐ups), and 25 μm (f, g).

We applied this workflow to sections of Arabidopsis siliques and leaves, *Camelina sativa* and *Solanum lycopersicum* (tomato) leaves (Figure 6b–g). In Arabidopsis siliques, AO and FY revealed the seed surface and endosperm‐associated cuticles, in addition to exocarpic and endocarpic cuticle‐like structures (Figure 6b,c). Additionally, AO signal was detected in silique valves, consistent with previously described endocarp b cell lignification (Nichol & Samuel, 2024; Roeder & Yanofsky, 2006) (Figure 6b). Sectioned *Camelina sativa* premature and mature green stage seeds stained with AO or FY confirmed the deposition of suberin as observed in the whole seeds (Figures 3i and 6d,e; Figure S5b).

Beyond reproductive tissues, the workflow was readily adapted to leaf samples. In both Arabidopsis and tomato, sectioning‐assisted clearing enabled fast visualization of the surface cuticle using AO and FY, respectively (Figure 6f,g). Notably, the complete workflow could be performed within 24 h for leaf samples and within 1–2 days for siliques and *Camelina sativa* seeds, providing a rapid approach for imaging barrier structures in otherwise inaccessible tissues. Together, these results demonstrate that vibratome‐assisted optical clearing extends the applicability of the method beyond intact seeds and provides a versatile platform for resolving the spatial organization of cuticle‐ and suberin‐based barriers in complex plant organs.

## DISCUSSION

Visualizing lipid‐based extracellular barriers within intact seeds remains challenging because these structures are embedded within optically dense and highly compartmentalized tissues. The optimized clearing and staining workflow presented here overcomes many of these limitations and enables systematic visualization of the major seed barrier domains, including the seed surface cuticle, seed coat suberin, the endosperm‐associated cuticle, and the embryo cuticle. Beyond providing improved optical access, this approach allows these structures to be analyzed within a common spatial framework, highlighting the multilayered organization of seed barriers extending from the environment to the embryo. By complementing previous studies of individual barrier domains, the workflow enables their relative spatial organization, developmental progression, and remodeling to be compared under common experimental conditions across successive developmental stages.

A major advantage of the workflow is the combination of whole‐mount imaging, rapid sample processing, and applicability across successive developmental stages. Previous histological and ultrastructural studies have provided detailed information on individual seed barriers, but these structures have generally been investigated separately and using different experimental approaches. The present workflow complements these studies by enabling multiple barrier domains to be examined under common experimental conditions while preserving their spatial context within intact seeds. Its compatibility with fluorescent reporters further allows barrier organization to be related to the spatial activity of genes associated with specific cell identities or barrier biosynthesis. The developmental series presented here illustrates this capacity through visualization of the late accumulation of seed coat suberin, consistent with previous observations (Hyvärinen et al., 2025), together with changes in seed surface cuticle organization associated with mucilage deposition.

The developmental patterns observed across the different barrier domains also generate new biological hypotheses. Seed coat suberin showed a clear developmental onset, being undetectable in the oi1 layer at early stages and accumulating during late seed development to form a continuous suberized domain. This developmental progression is reminiscent of the establishment of suberin in the Arabidopsis root endodermis, where suberization progressively increases as the tissue matures (Andersen et al., 2015; Barberon et al., 2016). Although the spatial and developmental contexts of these two barriers are distinct, both illustrate the progressive establishment of a suberin‐rich apoplastic domain. Whether this developmental accumulation of seed coat suberin involves regulatory principles similar to those controlling endodermal suberization remains an interesting question for future investigation.

The relatively late establishment of continuous seed coat suberization, when considered alongside the earlier presence of cuticular barriers, also raises the possibility that distinct barrier systems contribute differentially to seed protection at successive developmental stages. Beyond acting as diffusion barriers, extracellular seed structures can also perform specialized mechanical functions. The endosperm‐derived embryo sheath, for example, acts as an anti‐adhesive layer that facilitates cotyledon emergence during germination (Doll et al., 2020), while the seed surface cuticle and mucilage are thought to influence seed hydration and interactions with the external environment. Comparing these structures within a common developmental framework may therefore help investigate how multiple extracellular matrices contribute to seed maturation and germination. Imaging of the seed surface revealed dynamic changes in the organization of the outer cuticle during mucilage deposition and extrusion. The apparent redistribution and reduction in cuticle‐associated labelling during this process suggest a close developmental relationship between cuticle organization and mucilage secretion, a phenomenon that remains poorly understood and merits further investigation. More broadly, the ability to visualize surface cuticle dynamics, seed coat suberization, and the embryo‐ and endosperm‐associated cuticles within a common experimental framework provides an opportunity to investigate their spatial and developmental relationships across maternal and zygotic seed tissues.

Whole‐mount imaging also provides information complementary to conventional histological and ultrastructural approaches by preserving the spatial continuity and three‐dimensional organization of barrier domains within intact seeds. This is particularly relevant for barriers that display spatial heterogeneity during their establishment or remodeling, as individual cross‐sections provide information from defined planes whose interpretation depends on sectioning position and orientation. For example, our developmental series revealed a suberin deposition gradient along the oi1 layer during seed maturation, allowing the spatial progression of seed coat suberization to be followed.

Three‐dimensional reconstruction of the endosperm‐associated cuticle at the chalazal region further illustrates the ability of whole‐mount imaging to resolve barrier organization around structurally complex seed interfaces without physical sectioning. Similarly, imaging larger areas of the seed surface revealed changes in the organization of the outer cuticle associated with mucilage deposition and extrusion. Thus, rather than replacing section‐based histological or ultrastructural approaches, whole‐mount imaging provides a complementary means of examining barrier continuity, spatial heterogeneity, and developmental progression within the intact seed.

Despite these advantages, several limitations should be considered. The fluorescent dyes used here differ in their specificity and may label overlapping classes of lipid‐rich extracellular structures. In addition, optical clearing and staining procedures may alter the integrity of highly exposed barrier domains, particularly at the seed surface. Although the workflow provides high‐resolution three‐dimensional information, it does not replace ultrastructural approaches such as transmission electron microscopy, which remain essential for resolving fine‐scale barrier architecture. Consequently, the methods presented here should be viewed as complementary to ultrastructural and biochemical analyses.

Beyond developing Arabidopsis seeds, the compatibility of the workflow with Arabidopsis siliques, Camelina seeds and tomato leaves, suggests its potential applicability to other species and organ systems. The integration of vibratome‐assisted sectioning further expands its utility to tissues that remain challenging for conventional whole‐mount clearing approaches. Together, these advances establish a versatile imaging platform for investigating the spatial organization and developmental progression of cuticle‐ and suberin‐based barriers. By enabling multiple barrier domains to be examined under comparable experimental conditions across successive developmental stages, the approach provides a foundation for future studies aimed at understanding how extracellular diffusion barriers contribute to seed physiology, environmental adaptation, and plant resilience.

## EXPERIMENTAL PROCEDURES

### Plant material and constructs

*Arabidopsis thaliana* ecotype Col‐0 was used in this study as a genetic background. All seeds were sown in individual pots and stratified for 2 days at 4°C in the dark, then transferred to 16 h/8 h light/dark condition greenhouse chamber. Seeds were collected from 24‐ to 30‐day‐old plants for fixation and consecutive clearing and staining. Reporter lines *pGPAT5::NLS‐3xVenus* and *pATML1::NLS‐3xVenus* were generated by PCR amplifying the promoters of *GPAT5 (AT3G11430)* and *ATML1 (AT4G21750)* using the primers indicated in Table S1. Amplified promoters were introduced into an entry Gateway clone containing attL4‐attR1 Gateway recombination sites. The resulting entry clones containing *pGPAT5* and *pATML1* promoters were recombined with *NLS‐3xVenus* into a final T‐DNA Gateway destination vector, containing fluorescent seed (*pFG24GW*) selectable marker for plants (https://www.addgene.org/133747/).

### Fixation procedure

The precise information on seed fixation is provided in Methods S1. In short, fixation was adapted from the protocol described in Attuluri *et al*. (2022). Seeds were collected in 5 mL Eppendorf tubes containing freshly prepared 4% paraformaldehyde in 1X PBS‐T. Samples were then placed on a rotating wheel at 4°C for 15 min to ensure that seeds are in contact with the fixative solution before undergoing 1–2 h of vacuum infiltration on ice, followed by overnight incubation at 4°C. The fixative solution was washed twice with PBS‐T for 1 h each at 4°C with constant agitation then rinsed with PBS‐T before clearing procedure.

### Clearing and staining procedure

The protocols developed in this study were adapted from previously described ClearSeeα‐ and methanol‐based clearing procedures (Sexauer et al., 2021; Ursache et al., 2018; Ursache et al., 2021). To improve reagent penetration and reduce processing times, fixation and clearing steps were combined with vacuum infiltration and temperature‐controlled incubations. Depending on developmental stage and tissue type, samples were cleared at room temperature, 37°C, or 60°C and subsequently stained with NR, AO, FY, SR2200, or Calcofluor White (CW). Additional protocol modifications included optimized washing procedures for FY staining and mounting of cleared samples in a thin layer of soft agarose within chambered cover glass slides to improve sample stability during imaging. Detailed protocols for fixation, clearing, staining, washing, and mounting are provided in Methods S1. Samples were imaged using Olympus FV1000 or Zeiss LSM 980 confocal microscopes. CW or SR2200 was excited at 405 nm and detected at 420–455 nm. FY was excited at 488 nm and detected at 499–543 nm, AO was excited at 445 nm and detected at 482–543 nm, NR was excited at 561 nm and detected at 561–632 nm, and Venus's fluorescence was excited at 514 nm and detected at 526–543 nm.

### Vibratome sectioning

To visualize cuticle and suberin barriers using vibratome sectioning, Arabidopsis siliques, leaves, tomato leaves, and Camelina seeds were placed in melted 8% (w/v) agarose in 50 mL Falcon tubes and rapidly aspirated with a decapitated 5 mL syringe, whereas rolled leaves using a 20 decapitated mL syringe. Blocks were cooled on ice for 10 min and trimmed prior to sectioning. Sections (200 μm thickness) were generated using a Leica VT1000S vibratome. Sections were subsequently fixed in 4% (w/v) paraformaldehyde (PFA) in PBS‐T for 1 h, cleared in ClearSeeα supplemented with SR2200 for 24 h, and stained for 12 h with ClearSeeα‐compatible dyes, including AO or ethanol‐dissolved FY for Arabidopsis siliques and Camelina seeds. Arabidopsis leaf sections were fixed for 1 h in 4% (w/v) PFA in PBS, cleared in ClearSeeα for 24 h, then stained with AO and CW for 24 h. tomato leaf sections were fixed and cleared for 24 h in pure methanol before staining with methanol‐dissolved Calcofluor white and FY sequentially for 1 h each. Samples were mounted in chambered cover glass slides in a thin layer of soft agarose and imaged using a Zeiss LSM 980 confocal microscope under the conditions described in Methods S1.

## Conflict of Interest

The authors declare no conflicts of interest.

## Supporting information


**Figure S1.** Procedure for mounting seed samples. (a) Materials used for sample mounting. (b) Collection of stained and washed seeds. (c) Deposition of the seeds into the chamber while gently tilting it. (d) Cutting a small piece of 0.3% agarose using a ruler. (e) Close‐up view of the agarose piece. (f) Placement of the agarose piece over the seeds using a brush. (g) Mounted seed samples ready for microscopy.
**Figure S2.** Detection of Arabidopsis seed apoplastic barriers using fluorescent dyes for cuticle visualisation. Seeds stained with Nile Red (a–e), Auramine O (f–j) and Fluorol Yellow (k–o). Yellow, blue, green, pink and white arrows indicate the seed surface, endosperm‐associated and embryo cuticles and seed coat suberin, respectively. Scale bar: 500 μm (a, f, k) and 100 μm (b, e, g–j, m–o).
**Figure S3.** Outer integument 2 cell wall and barrier remodelling dynamics during seed development. (a–j) Representative images of developing Arabidopsis seeds at 3 DPA (a), 5 DPA (b), 7 DPA (c) and 10 DPA (d), cleared with Clearseeα of at 60°C and stained with Nile Red and SR2200. (e, f) Close‐up views of outer integuments from the regions indicated in (a–d). (i) Representative images illustrating seed surface cuticle stability following staining with Clearseeα‐dissolved AO or NR. (j) Bar plot showing the progressive stage‐dependent destabilization of the seed surface cuticle. Number of samples observed: N_Globular ‐ Linear_ = 26, N_Late linear ‐ Bending_ = 24, N_Mature green_ = 15. Yellow, blue and pink arrows indicate the seed surface cuticle, endosperm‐associated embryo cuticle, and seed coat suberin, respectively. oi: outer integument; ii: inner integument; col: columella. Scale bar: 100 μm (a–d), 50 μm (e–h) and 25 μm (i).
**Figure S4.** Visualization of apoplastic barriers in dry and developing *Arabidopsis* seeds. (a–d) 3D reconstruction of regions of dry Arabidopsis seed stained using the methanol‐based Nile Red staining protocol. (a, b) The dry seed outer integument reveals shows remain of the seed surface cuticle as veil‐like structures (red arrow) suspended above the strongly stained seed coat suberin (purple arrow). (c, d) The suberized outer integument (oi1), labelled by Nile Red, is visible beneath the extruded mucilage of outer integument (oi2), labelled by SR2200. (e, f) *Arabidopsis thaliana* premature (3 DPA; e) and mature green (10 DPA; f) seeds stained with phenolic cell wall component‐associated dye Basic Fuchsin. Blue, orange and white arrows indicate the seed surface, endosperm associated and outer integument 1 phenolic barrier, respectively. Scale bar: 10 μm (a, b), 30 μm (c, d) and 100 μm (e, f).
**Figure S5.** Visualization of apoplastic barriers in developing *Camelina sativa* seeds. Heart‐stage (a) and mature green‐stage (b) Camelina seed cleared and stained with Auramine O. Yellow, green and pink arrows indicate the seed surface cuticle, embryo cuticle and seed coat suberin, respectively. Scale bar: 100 μm, close‐ups: 50 μm.
**Figure S6.** 3D reconstruction of Arabidopsis 3 DPA seed chalazal region show endosperm cuticle opening. (a–d) Representative images of the chalazal region of a premature seed (a, b). (c, d) Close‐up views of the chalazal region revealing an opening in the endosperm‐association cuticle. Samples were stained with Nile Red (a–d) and SR2200 (a). Scale bar: 50 μm.
**Figure S7.** Detailed procedure for sample embedding and sectioning. (a–e) Materials used for sample molding and embedding. (a) A 50 mL Falcon tube cut in half for casting agarose blocks. (b, c) Cut syringes of different sizes used to hold and mold samples according to sample size. (d) Metal mold used for embedding Camelina seeds. (e) Materials used to roll leaves and maintain them in a rolled configuration before embedding. (f) General overview of the vibratome sectioning workflow.
**Methods S1.** Detailed protocol for whole‐seed barrier visualization using histological staining in combination with fluorescent protein reporters.
**Table S1.** Primers used for promoter amplification and transcriptional reporter construction.

## Data Availability

All data supporting the findings of this study are included in the article and its Supporting Information.
