# Supplementary material for: Bringing seed barriers into focus: Optimized clearing and histological staining
Source: Plant J. 2026 Nov 3;128(3):e71209. doi: 10.1111/tpj.71209 (PMC13728890; doi:10.1111/tpj.71209)
Supplement: Supplementary file 1 [file TPJ-128-0-s001.pdf]

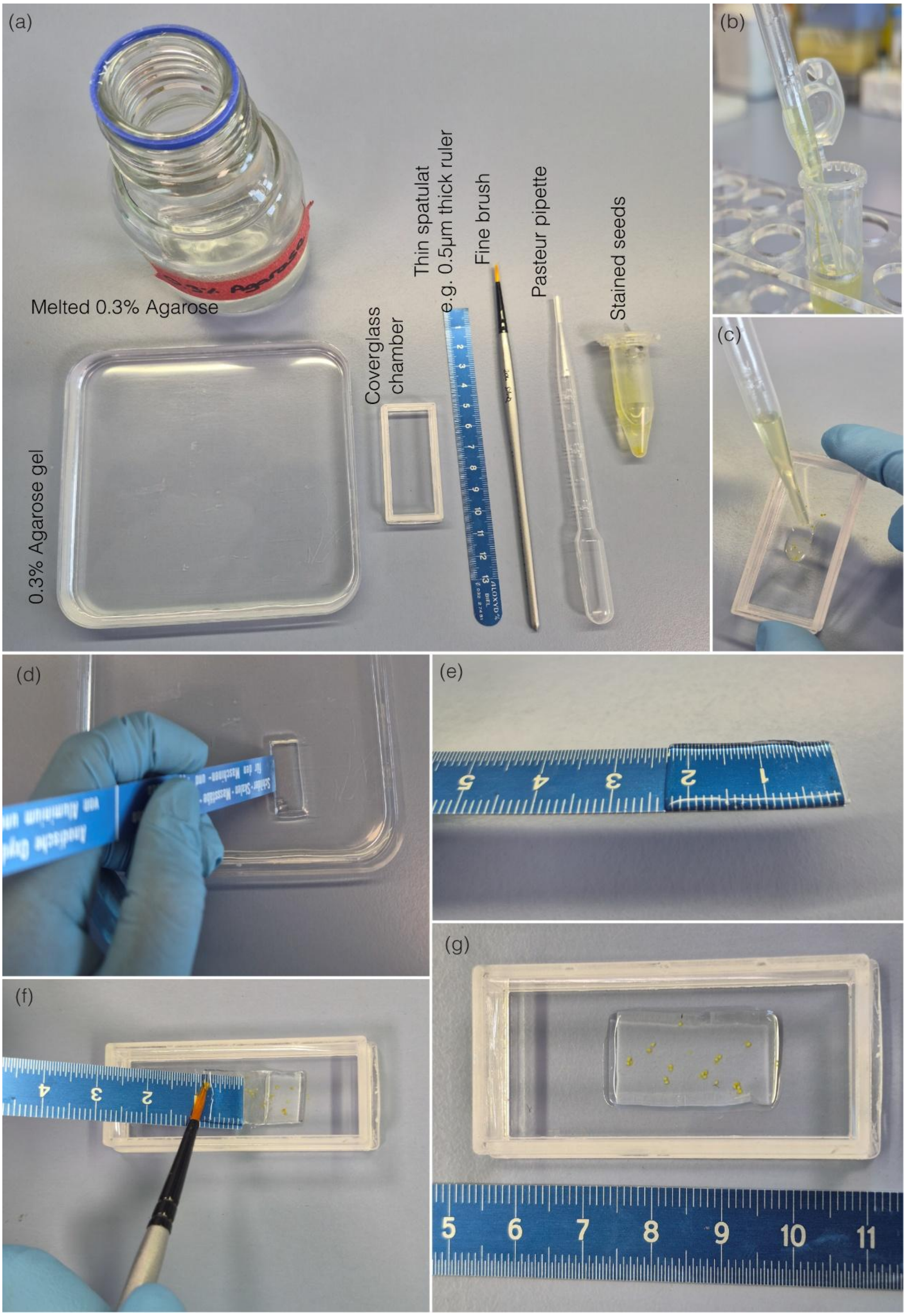

Figure S1: Procedure for mounting seed samples. (a) Materials used for sample mounting. (b) Collection of stained and washed seeds. (c) Deposition of the seeds into the chamber while gently tilting it. (d) Cutting a small piece of 0.3% agarose using a ruler. (e) Close-up view of the agarose piece. (f) Placement of the agarose piece over the seeds using a brush. (g) Mounted seed samples ready for microscopy.

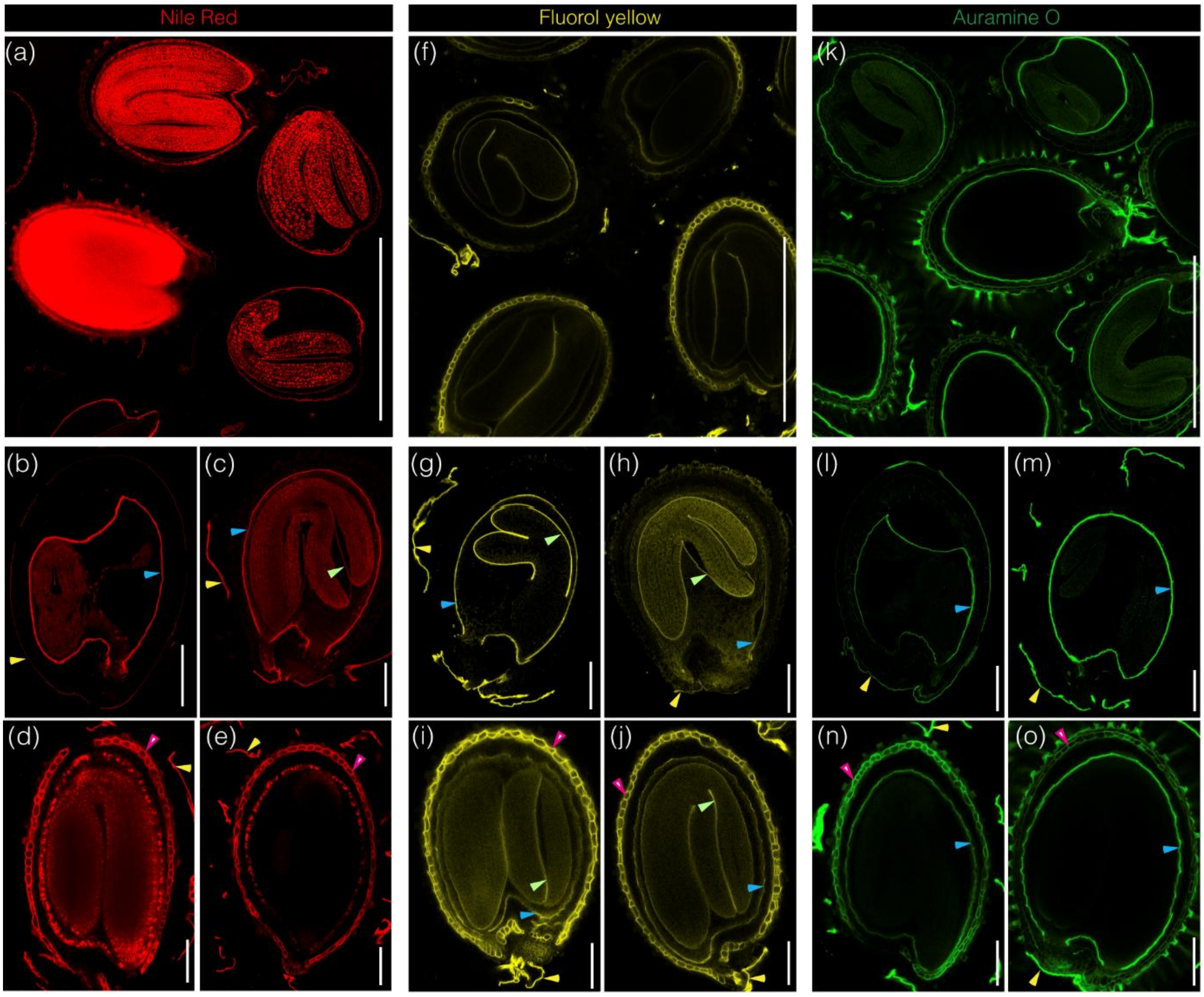

Figure S2: Detection of Arabidopsis seed apoplastic barriers using fluorescent dyes for cuticle visualisation. Seeds stained with Nile Red (a-e), Auramine O (f-j) and Fluorol Yellow (k-o). Yellow, blue, green, pink and white arrows indicate the seed surface, endosperm-associated and embryo cuticles and seed coat suberin, respectively. Scale bar: 500µm (a, f, k) and 100µm (b-e, g-j, m-o).

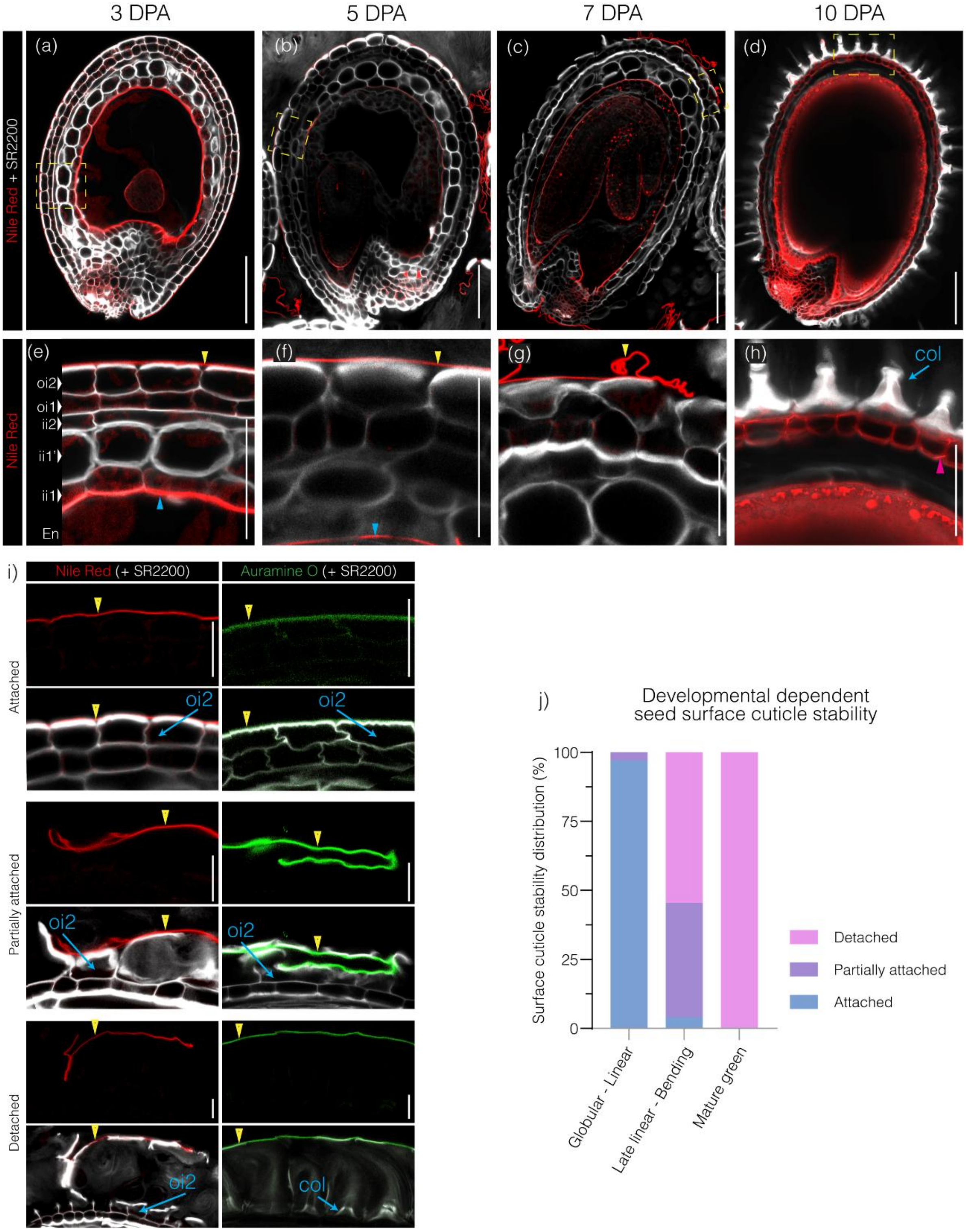

Figure S3: Outer integument 2 cell wall and barrier remodelling dynamics during seed development. (a-j) Representative images of developing Arabidopsis seeds at 3 DPA (a), 5 DPA (b), 7 DPA (c) and 10 DPA (d), cleared with Clearseeα of at 60°C and stained with Nile Red and SR2200. (e-f) Close-up views of outer integuments from the regions indicated in (a-d). (i) Representative images illustrating seed surface cuticle stability following staining with Clearseeα-disolved AO or NR. (j) Bar plot showing the progressive stage-dependent destabilization of the seed surface cuticle. Number of samples observed: $N_{Globular - Linear}$=26, $N_{Late\ linear - Bending}$=24, $N_{Mature\ green}$=15. Yellow, blue and pink arrows indicate the seed surface cuticle, endosperm-associated embryo cuticle, and seed coat suberin, respectively. oi: outer integument; ii: inner integument; col: columella. Scale bar: 100µm (a-d), 50µm (e-h) and 25µm (i).

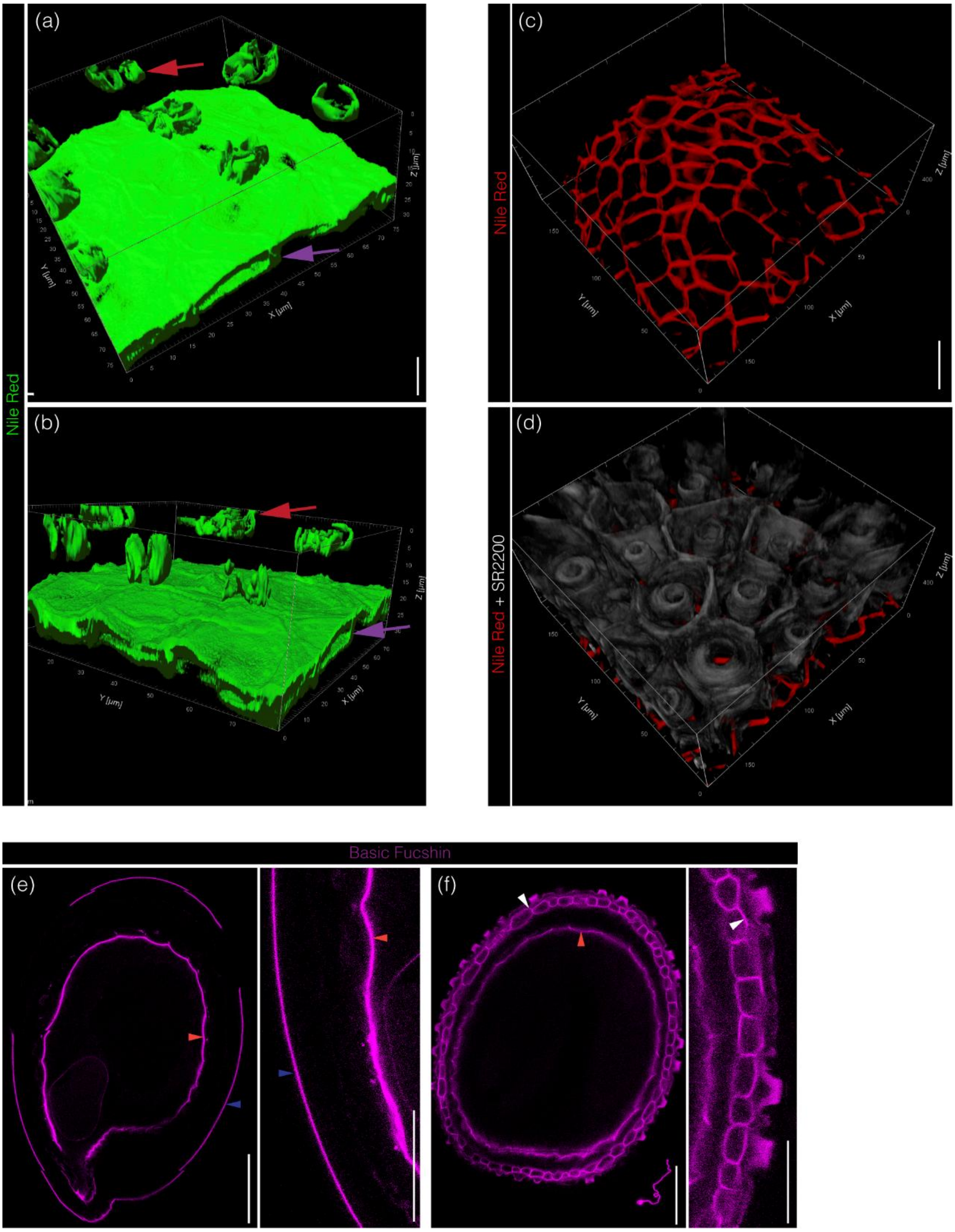

Figure S4: Visualization of apoplastic barriers in dry and developing *Arabidopsis* seeds. (a-d) 3D reconstruction of regions of dry Arabidopsis seed stained using the methanol-based Nile Red staining protocol. (a-b) The dry seed outer integument reveals shows remain of the seed surface cuticle as veil-like structures (red arrow) suspended above the strongly stained seed coat suberin (purple arrow). (c-d) The suberized outer integument (oi1), labelled by Nile Red, is visible beneath the extruded mucilage of outer integument (oi2), labelled by SR2200. (e-f) *Arabidopsis thaliana* premature (3 DPA; e) and mature green (10 DPA; f) seeds stained with phenolic cell wall component-associated dye Basic Fuchsin. Blue, orange and white arrows indicate the seed surface, endosperm associated and outer integument 1 phenolic barrier, respectively. Scale bar: 10µm (a, b),30µm (c, d) and 100µm (e, f).

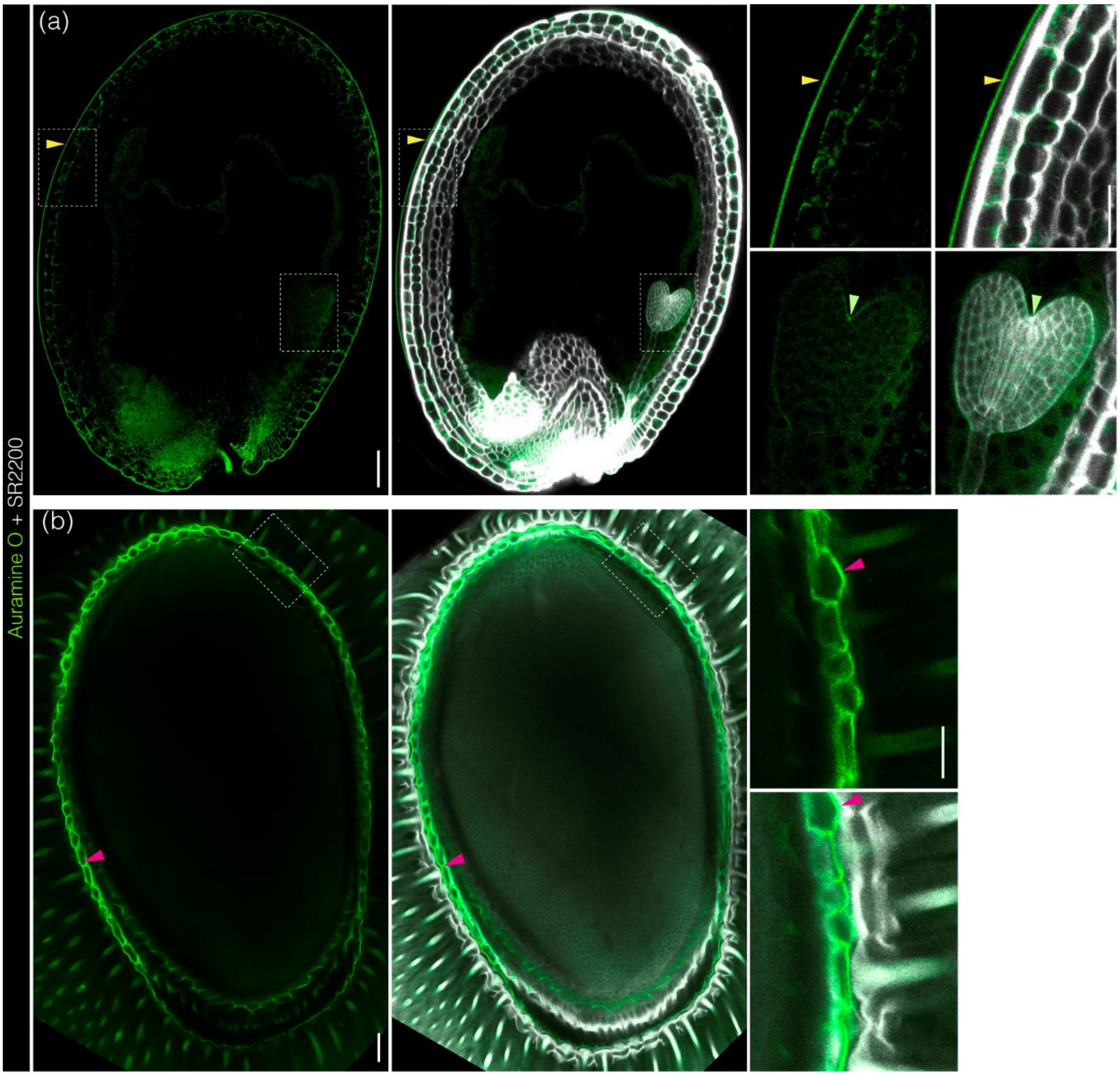

Figure S5: Visualization of apoplastic barriers in developing *Camelina sativa* seeds. Heart-stage (a) and mature green-stage (b) Camelina seed cleared and stained with Auramine O. Yellow, green and pink arrows indicate the seed surface cuticle, embryo cuticle and seed coat suberin, respectively. Scale bar: 100µm, close-ups: 50µm.

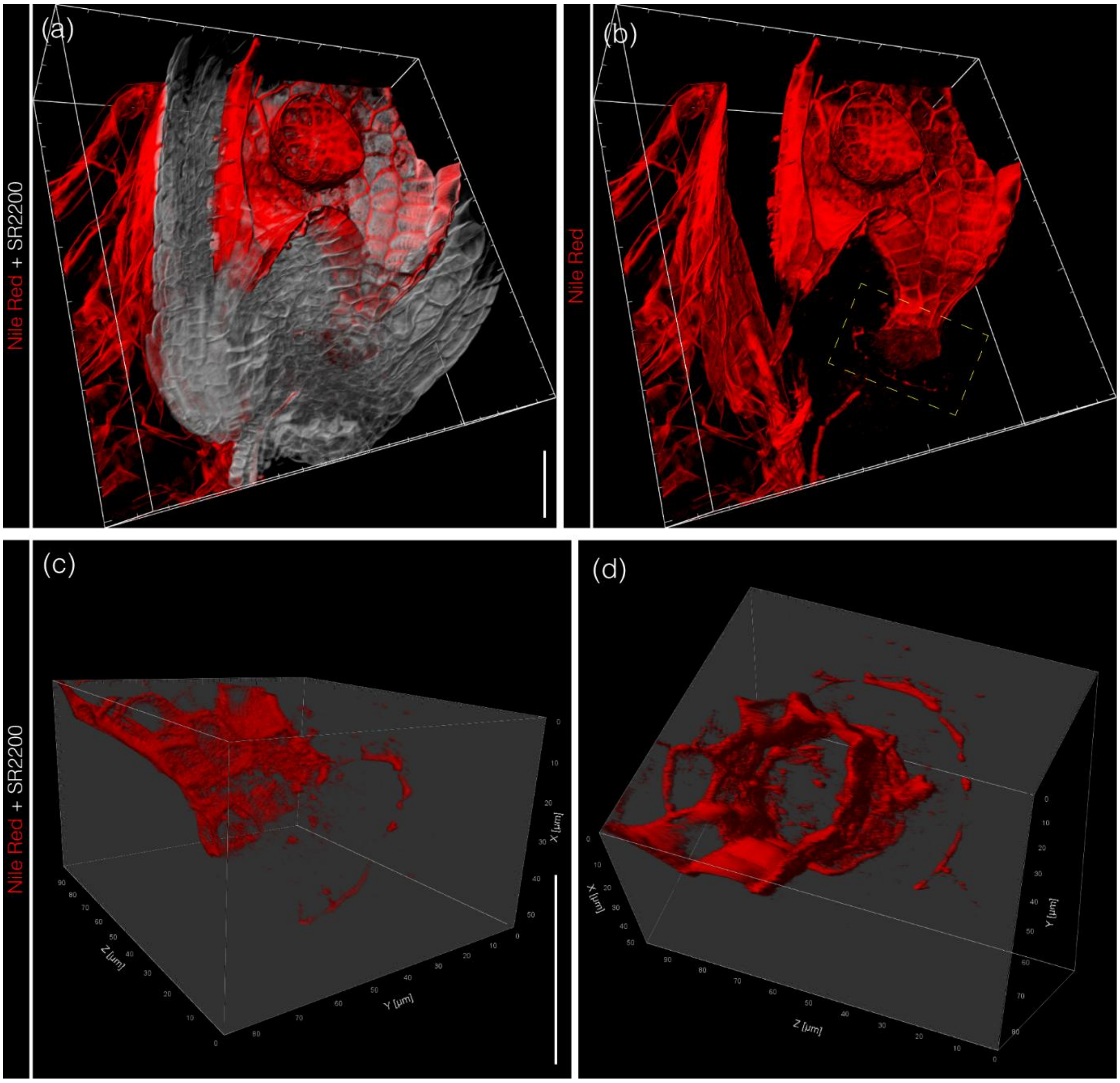

Figure S6: 3D reconstruction of Arabidopsis 3 DPA seed chalazal region show endosperm cuticle opening. (a-d) Representative images of the chalazal region of a premature seed (a, b). (c,d) Close-up views of the chalazal region revealing an opening in the endosperm-association cuticle. Samples were stained with Nile Red (a-d) and SR2200 (a). Scale bar: 50µm.

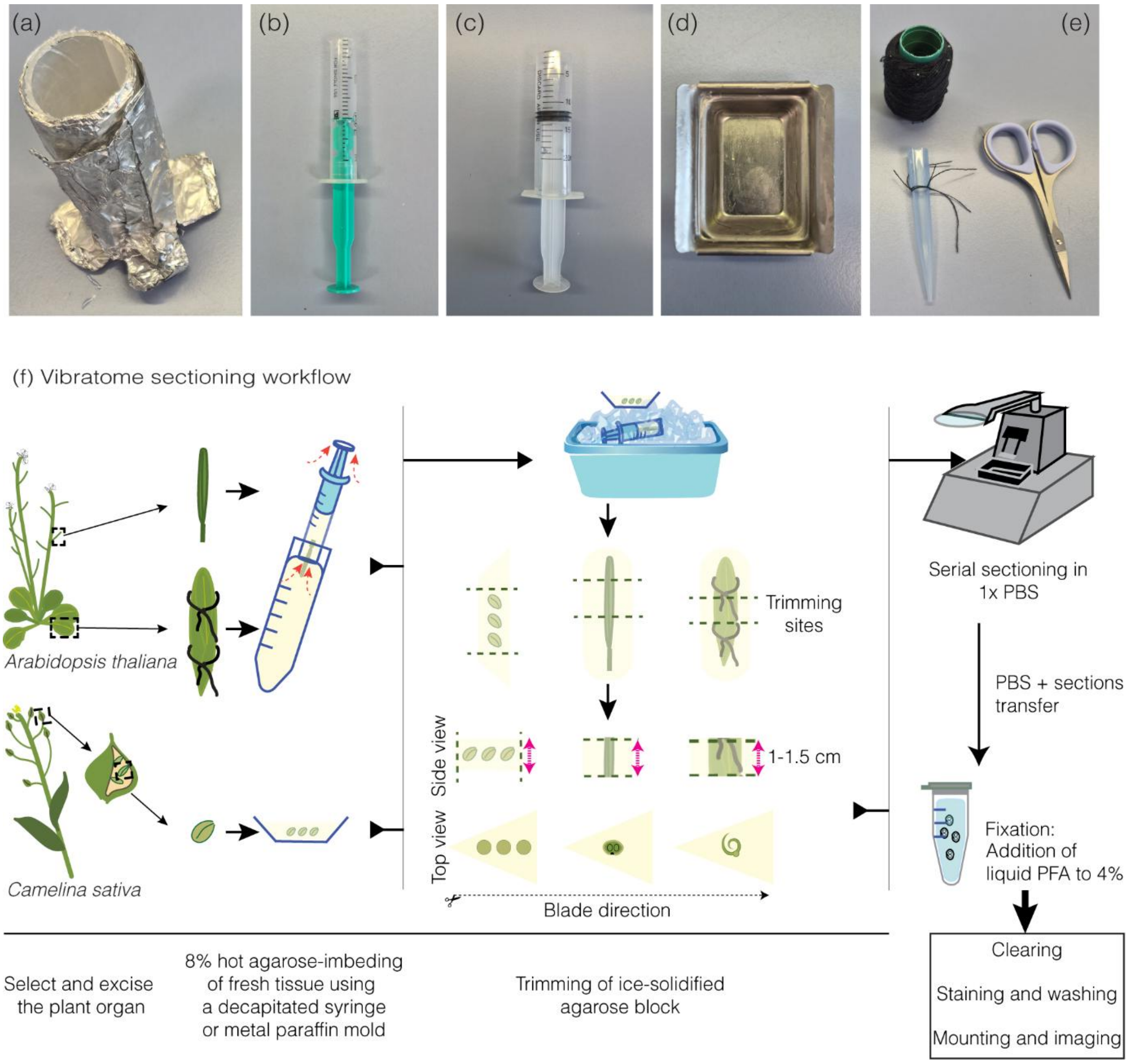

Figure S7: Detailed procedure for sample embedding and sectioning. (a-e) Materials used for sample molding and embedding. (a) A 50 ml Falcon tube cut in half for casting agarose blocks. (b, c) Cut syringes of different sizes used to hold and mold samples according to sample size. (d) Metal mold used for embedding Camelina seeds. (e) Materials used to roll leaves and maintain them in a rolled configuration before embedding. (f) General overview of the vibratome sectioning workflow.

Bringing seed barriers into focus: optimised clearing and histological staining

Nirina et al,

CHEMICALS:

The following chemicals were used to develop the protocol: PFA (Paraformaldehyde) (Merck, CAS-No: 30525-89-4), 32% PFA aqueous solution (EMS, CAS-No: 50-980-494), Triton-X100 (Carl Roth, CAS-No: 3051.2), Xylitol (Sigma, CAS-No: 87-99-0), Sodium Deoxycholate (Sigma, CAS-No: 302-95-4), Urea (Sigma, CAS-No: 57-13-6), Sodium sulfite (ThermoScientific, CAS-No: 7757-83-7), SCRI Renaissance 2200 (SR2200) (Renaissance Chemicals Ltd, https://renchem.co.uk/products/renaissance-SCRI-2200), Calcofluor White M2R (Fluorescent brightener 28) (Polysciences, CAT#4359), Nile Red (Sigma, CAS-No: 7385-67-3), Auramine O (Sigma, CAS-No: 2465-27-2), Fluorol Yellow 088 (Santa Cruz Biotechnology, CAS-No: 81-37-8), Agarose (Merck, CAS-No: 9012-36-6), and Coverglass chamber (ThermoScientific, CAS-No: 155360). Optimal results were achieved using the chemicals purchased from the above-mentioned suppliers.

RECIPES

- **4% PFA (PARAFORMALDEHYDE) PREPARATION**

Prepare 4% PFA by diluting 32% PFA commercial solution in 1X PBS:0.05% Triton 100X or from powdered PFA as follow:

Dissolve 4g of powder paraformaldehyde in 100mL 1X PBS by:

- Heating to 50°C while stirring. **Don't exceed 65**°C!
- The powder will not immediately dissolve. Raise the pH to 10 by adding NaOH dropwise and leave the solution to clear in about 5 minutes.
- Once the paraformaldehyde is dissolved, adjust the pH down to 7.2 by adding HCl dropwise.
- Let it slowly cool down to room temperature then add 100X Triton up 0.05% final concentration.

Place on ice for at least 30min before use or store at 4°C.

NOTE: *Try to use always fresh PFA solution and store it in 4℃ for a week maximum.*

- **CLEARSEEα SOLUTION (Kurihara et al., 2021):**

XYLITOL [final 10% (w/v)] (Sigma),

SODIUM DEOXYCHOLATE [final 15% (w/v)] (Sigma)

SODIUM SULFITE [final 0.63% (w/v)] (Sigma)

UREA [final 25% (w/v)] (Sigma)

WATER to the final volume

Mix the solution vigorously by shaking and leave overnight on the magnetic stirrer until completely dissolved. Clearseeα can be stored at room temperature.

A-CLEARSEEα COMPATIBLE PROCEDURES

FIXATION:

- Expose seeds from the siliques by removing the valves with precision tweezers.
- Collect the seeds (or replum with seeds attached) in 2mL Eppendorf tubes (no more than 3-4 siliques worth of seeds per tube) containing 4% PFA in 1 x PBS-T.
- Vacuum-fix the samples in **4% PFA** in 1 x PBS-T for 1 hour on ice.
  Tip1: Apply vacuum for 15 minutes, slowly release the vacuum, invert the tubes 4-6 times, wait 15 minutes. Repeat once more.
- Place Eppendorf tubes on a rotating wheel overnight at 4°C.
- Wash twice with 1X PBS-T for 1 hours each, then rinse once in 1X PBS-T before clearing.

CLEARING:

- Replace 1X PBS-T with Clearseeα supplemented with **1% SR2200** or **0.1% Calcofluor White (CW)** and apply vacuum treatment for 2 hours.
- *Tip1: Apply vacuum for 15 minutes, slowly release the vacuum, invert the tubes 4-6 times, wait 15 minutes. Repeat once more.*
- Replace the solution with fresh Clearseeα with **SR2200** or **CW**.
- Incubate at 60°C, 37°C or at room temperature following the instructions provided below:

Incubation temperature	Pre-globular to linear stage	Bending cotyledon stage	Mature green
60°C	*>12h*	*>36h*	*>15days*
37°C	*>48h*	*>96h*	*>20days*
Room temperature	*>15days*	*>1.5month*	*X*

- Image **SR2200** or **CW** with 405 nm excitation and detect at 420-455 nm.

STAINING PROCEDURES

1. Auramine O staining of seed cuticles and seed coat suberin

- Prepare 0.5% solution of **Auramine O** directly in ClearSeeα.
- Stain for 12-16 hours for young seeds and 36-48h for mature green at 37°C.
- Remove all **Auramine O** solution and rinse twice in ClearSeeα.
- Wash in ClearSeeαfor 30 min at 37°C, then mount on chambered coverglasses for imaging.

  Note: *Extend washing step to at least 4 h for mature seeds and complex tissue*.
- Image **Auramine O** with 445 nm excitation and detect at 482-543 nm.

2. Nile Red staining of seed cuticles and seed coat suberin

- Prepare 0,05 % solution of **Nile Red** directly in Clearseeα.

  Note: *Filter staining solution before use.*
- Stain for 12-16 hours for young seeds and 36-48h for mature green at 37°C.
- Remove all **Nile Red** solution and rinse once in ClearSeeα.
- Wash in ClearSeeα for 30 min at 37°C, then mount on chambered coverglasses for imaging.

  Note: *Extend washing step to at least 4 h for mature seeds and complex tissue.*
- Image **Nile Red** with 561 nm excitation and detect at 562-632 nm.

3. Fluorol yellow staining of seed cuticles and seed coat suberin

- Prepare 1% stock solution of **Fluorol yellow** in DMSO by heating it at 37°C for at least 10 minutes while vigorously shaking. Store the stock solution at 4°C.
- Prepare a 0.01% FY working solution by diluting the stock solution in 96% ethanol.
- Stain for 12-16 hours at 37°C regardless of developmental stage.
- Replace the staining solution with 75% Ethanol and mix 4-6 times by inverting the tube.
- Let the seeds sink (5-10 minutes) and apply sequential (5-10 minutes) ethanol steps - 50%, 25% and wash twice with water before mounting on coverglass chambers.
- Image **Fluorol yellow** with 488 nm excitation and detect at 499-543 nm.

B- NON-CLEARSEEα COMPATIBLE PROCEDURES

1. Methanol-based fixation, clearing and Fluorol yellow staining of seed surface cuticle

- Expose seeds from the siliques by removing the valves with precision tweezers.
- Collect the seeds (or replum with seeds attached) in 2mL Eppendorf tubes (no more than 3-4 siliques worth of seeds per tube) containing pure methanol.
- Refresh the methanol when the solution becomes green.
- After 3 days, stain with 0.01% **Fluorol yellow** dissolved in methanol for 12-16 hours regardless of developmental stage.
- Rinse briefly 3 times with methanol, then mount on chambered coverglasses for imaging.
- Image **Fluorol yellow** with 488 nm excitation and detect at 499-543 nm

2. Methanol-based fixation, clearing and Nile red staining of mature dry seed coat suberin

- Put around 50-100 mature dry seeds in 2mL Eppendorf tubes containing pure methanol.
- Refresh the methanol frequently (daily) during at least 1 week until the solution remains clear.
- After 1 week, stain with syringe-filtered 0.05% **Nile red** dissolved in methanol for 12-16 hours.
- Rinse briefly 3 times with methanol, then mount on chambered coverglasses for imaging.

  Image **Nile Red** with 561 nm excitation and detect at 562-632 nm.

SAMPLE MOUNTING

- Carefully collect the replum using the paint brush and loose individual seeds with Pasteur pipette from the 2mL Eppendorf tubes.
- Place the replum or the seeds directly on the bottom of the coverglass chamber.

  Note: *tilting the chamber is recommended while placing the seeds with the Pasteur pipette to retain the seeds at the surface of the coverglass.*

- Remove the excess fluid from the chamber.
- Scoop up a thin layer of 0.3% Agarose gel with a thin spatula.
- Gently slide the gel off the spatula with a brush to place the seeds between the agarose gel and the coverglass as shown in the Supplemental Figure S1.

  Tip1: Coverglass is easily replaceable by gluing a microscopy coverslide in its stead.

NOTE:

- **SR2200** or **CW** and **Nile Red** stained seed are compatible with ***NLS-3xVenus*** marker lines. For **Nile Red** use 561 nm excitation and 562-632 nm emission, for **SR2200** or **CW** use 405 nm excitation and 420-455 nm emission, for ***NLS-3xVenus*** 514 nm excitation and 526-543 nm emission.

Table S1: Primers used for promoter amplification and transcriptional reporter construction.

Promotor name (TAIR ID)	Forward (5'-3') AttL4 complementary overhang in red	Reverse (5'-3') AttR1 complementary overhang in red
***pGPAT5* (AT3G11430)**	ATCAGATCTTCTAGAGGATCTGATCGCAAACGTCAATGGTCTATATCC	TTTGTACAAACTTGCGGATCTTCTTTTGTTTTTTGCTCGAATATTATTTTGACTCG
***pATML1* (AT4G21750)**	ATCAGATCTTCTAGAGGATCGGTCTCAACCTGGAACAACTAAGC	TTTGTACAAACTTGCGGATCGGTCTCTTGTTGATGATGATGGATGC
